# Liverwort flora of Ayan – a gained link between subarctic and hemiboreal floras in West Okhotiya (Pacific Russia)

**DOI:** 10.3897/BDJ.9.e65199

**Published:** 2021-04-01

**Authors:** Vadim Bakalin, Ksenia Klimova, Daniil Bakalin, Seung Se Choi

**Affiliations:** 1 Laboratory of Cryptogamic Biota, Botanical Garden-Institute of the Far Eastern Branch of the Russian Academy of Sciences, Vladivostok, Russia Laboratory of Cryptogamic Biota, Botanical Garden-Institute of the Far Eastern Branch of the Russian Academy of Sciences Vladivostok Russia; 2 AXiiO Oy Company, Helsinki, Finland AXiiO Oy Company Helsinki Finland; 3 Team of National Ecosystem Survey, National Institute of Ecology, Seocheon, South Korea Team of National Ecosystem Survey, National Institute of Ecology Seocheon South Korea

**Keywords:** diversity, phytogeography, Hepaticae, Northeast Asia, Dzhugdzhur Range

## Abstract

The liverwort flora of Ayan was first investigated one hundred and fifty years after the first exploration of vascular plants. A number of factors has determined the relatively high taxonomic diversity of liverworts in this hemiarctic flora of small-sized area: 118 species and one subspecies were revealed. These data are new not only for the studied area, but also for the huge land adjacent to the western coast of the Sea of Okhotsk. The liverwort flora possesses the domination of taxa common in the hemiarctic, although with a lot of taxa more common in boreal as well as arctic-alpine environments. The presence of Mega-Beringian and calciphilous taxa is the peculiar trait of the studied liverwort flora. Based on detrended correspondence analysis (DCA), Ayan liverwort flora shows relationships to the continental mainland floras situated both in North-East Asian hemiarctic and hemiboreal East Asia and is, therefore, the link between both. The flora of Ayan surroundings is one of the newly-filled ‘blank spots’ in the possible floral exchange way between Arctic Northeast Asia and mountainous floras of temperate East Asia.

## Introduction

The Russian-American Company under the High Patronage of His Imperial Majesty was founded in 1799 and urgently required new surface tracks to access the North Pacific from Europe via Siberia. The way from Yakutsk Town to the newly-founded Ayan Settlement at the coast of the Sea of Okhotsk was constructed and opened in 1844 and this was the only uninterrupted way in the middle of 19th century to access the Pacific Ocean by land across Eurasia (Lightfoot 2003). Fortunately for biologists, this way crossed a lot of geologically unique places with various climates and even the first indications by Regel and Tiling (1859) showed its richness and resulted in the description of several taxa bearing epithet ‘*ajanensis*’. Though some of such epithets were published even before that time, they appeared from the land adjacent south to the Ayan: i.e. *Picea ajanensis* Fisch. ex Carrière was described, based on materials collected by Middendorf in 1844 from the huge area “jugo Stanowoi … prope Udskoi … insula Schantar…” (=Stanovoy Range, Udskaya Bay, Shantar Islands) (von Trautvetter and Meyer 1856: 87) and does not belong to Ayan surroundings in the strict sense.

Thirty years after its establishment, the Ayan track was abandoned and was never established again as a summer-operated road, although some remnants of that monumental construction are still observable here and there along the Aldoma River. The closing of the track operation immediately stopped the investigations in the area for a long time. Then, the exploration of Ayan flora was continued in the second half of 20th century, after one hundred years (Khokhrjakov and Berkutenko 1979, Kharkevich et al. 1983, Schlothgauer 1990, Schlothgauer and Kryukova 2005). The exploration process was made possible to re-start and it was fruitful due to the wide use of helicopter transport as the only possibility to visit some of the more inaccessible places. The excellent materials collected at that time and reviewed later are confined to vascular plants, but did not include bryophytes. Although, in 1980-th, the bryologist Valentina Cherdantseva (see the description of her life and career in Bakalin 2013a) visited the Ayan Settlement and collected mosses, but those, unfortunately, were neither processed nor, certainly, published and apparently partially lost and partially stored unnamed in the VLA herbarium in Vladivostok. The collections of liverworts from that area are completely unknown to us. In the circumstances of potentially rich flora, the Ayan bryological "white spot" (Bakalin 2013b) existence looked very attractive. Indeed, its continuing existence is caused simply by the difficulty in accessing the site, due to the considerable distance from any federal car or rail roads (the nearest year-round operated road is 720 km west of Ayan in the Republic of Yakutia) and the only regular passenger communication operated from Khabarovsk City to Ayan by small aircrafts, which is extremely dependent on weather conditions. The latter is the only means at the disposal of the botanist who wants to visit Ayan in the summer season.

The geological diversity, described in the Material and Methods section, identifies high plant taxonomic diversity that may not be confined to vascular plants and likely appears in bryophytes.The high plant diversity in the area besides geology may be also foreshadowed by: 1) climate variation from subcontinental (locally suboceanic) in areas adjacent to the Sea of Okhotsk (also with frequent fogs shrouding the coast), to continental in the axial part of the Dzhugdzhur Range; 2) the sub-meridional stretch of the Dzhugdzhur Range axis linking the mountain flora of the south of eastern Siberia (the Stanovoy Range), which, in turn, is in contact with the mountains of East Asian Floristic Region, such as the Sikhote-Alin Mountain system. Northwards, the Dzhugdzhur Range contacts with the North-East Asia mountains, for example, the Kolyma Upland and Verkhoyansky Range. The listed factors determine the landscape diversity and influence community composition from the historical perspective and are a prerequisite for the discovery of a rich liverwort flora.

With specially supported field research in Ayan in 2019, we have visited this area and travelled from the sea coast to the ridge line in Dzhugdzhur Range collecting several hundred liverwort specimens within three weeks. The main goals of the present work are: 1) to describe the liverwort taxonomic diversity of this land; 2) to find the phytogeographical connections between floras in amphi-Pacific North-East Asia and 3) try to reveal the factors determining the diversity.

## Materials and Methods

### Study area

North Okhotiya is the informal name for a huge area of land surrounding North-West coast of the Sea of Okhotsk and geologically belongs to adjacent edges of the Eurasian and Pacific Geological Plates (Seno et al. 1996). If the geological map of the west coast of the Sea of Okhotsk is looked at, a mosaic of bedrocks of different composition and age would be very prominent at the 56th degree of the northern latitude, just southward of the contact zone of two plates (http://atlaspacket.vsegei.ru/#c9d3d204b97b3b163). This geological diversity ‘hot spot’ covers the vicinity of the Ayan Settlement and stretches westwards to the axial line of Dzhugdzhur Range – one of the great ranges orographically connecting Northeast Asia and East Asia. The rocky outcrops in this area vary from Archean time (observed mainly on the axial part of Dzhugdzhur Range), to younger rocks, up to the Cretaceous (in areas adjacent to the seacoast). This great age variation is combined with a variety of compositions: Archean rocks belonging to anorthosites, gabbroanorthosites, gabbros, gabbroamphibolites, norites, metagabbro, metapyroxenites, metaperidotites, various gabbroids, granites etc.; Proterozoic rocks represented by gabbros and dolerites; Paleozoic bedrocks represented by Devonian granites, granite-porphyry, diorites, diorite-porphyrite and gabbros. The youngest rocks are from the Mesozoic Cretaceous. Noticeable geological diversity may likely portend to a high taxonomic diversity in the flora.

The surroundings of Ayan is the area that is hard to access. In the vicinities of the settlement, there is only one all-year-round operated road of 12 km length that connects the settlement itself with the local airport. Another way goes to Nelkan Village, located 200 km west from Ayan and can be called the ‘road’ very conditionally, since it is passable only for three-axle heavy-wheeled vehicles and all-terrain (crawler) vehicles and only for two months per year (July and August), with the greater part of the track going directly by the riverbed. These circumstances make the exploration of the area very difficult and, before 1990-th, the works were mostly conducted using helicopter – a practice almost abandoned in the current floristic research in Russia. The maximal distance between localities studied in the present paper is about 60 km (by direct line). The studied localities were visited in the course of pedestrian routes combined with heavy vehicle transportation. The study was undertaken over a period of three weeks at the end of June and the beginning of July of 2019. In total ca. 400 specimens of liverworts and 350 specimens of mosses (not covered in the present account) were collected. The explored localities could be combined into three main areas:

1. The area immediately adjacent to the Ayan Settlement (3–7 km from the centre of the settlement);
2. Low and middle altitudinal belts of the northern part of Pribrezhnyi Range (Unych’ya River catchment);
3. Axial part of Dzhugdzhur Range, in other words, the watershed of the Pacific and Arctic Oceans, mainly in the upper reaches of the Tugorma River (more precisely, in one of its left tributaries).

**Figure 1.**
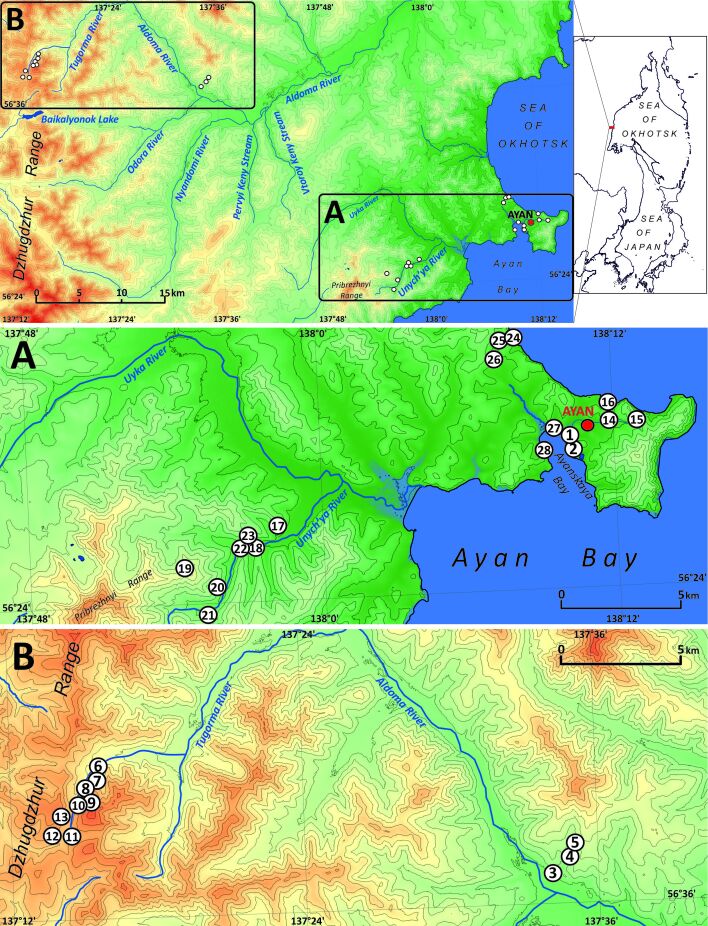
The collecting localities, numbered in accordance with Table 1.

For more details, the localities are shown in Table 1 and Fig. 1. The mosses were air-dried immediately, whereas the liverwort specimens were kept in the fridge (in anabiosis conditions) until they arrived at the Cryptogamic Biota laboratory in VBGI where they were identified alive by morphological methods and partly photographed (mostly for oil bodies data) and only then dried.

Since the main goal of the work was to reveal the liverwort diversity, the main efforts were assigned to their collection.. In the collection process, we tried to cover all types of vegetation communities and landscapes present in the studied area. In practice, this could not be achieved, due to both the inaccessibility of some geological formations and the time limit allocated for research. Nevertheless, we studied both presumed acidic rocks (meaning the pH of the water extract of the sedimentary products), for example granites, as well as presumed alkaline rocks, from various kinds of metamorphic rocks to limestone. Therefore, we expect the core of the liverwort flora to be revealed.

There are few data on the climate of Ayan surroundings. The weather station in Ayan Settlement provides data on the climate at low elevation. However, the climate parameters in the upper elevation and at the distance from the sea coast should be noticeably different. The Ayan weather station (https://en.climate-data.org/asia/russian-federation/khabarovsk-krai/ayan-718644/) is located at 24 m a.s.l., at a distance less than 500 m from the coast of Ayanskaya Bay of the Sea of Okthotsk. The mean annual temperature there is –3.26°C, the warmest month is August, as it commonly occurs in amphi-oceanic North Pacific areas (thus, not July), when the average temperature reaches 13.0°C, the coldest month is January with temperature average –19.6°C. The mean annual amount of precipitation is 856 mm per year, with a distinct late summer maximum from July to September, when the amount of precipitation exceeds 100 mm per month. The winter precipitation is quite scanty, represented by snow and does not exceed 33 mm per month from November to March. Taking account of available information, the Ayan surroundings climate may be called subarctic suboceanic that indeed corresponds to the dominance of subarctic vegetation in the coastal area, as described below. Going inwards from the Asian mainland, the climate transforms to continental, similar to that widely distributed in the south-eastern flank of the Republic of Yakutiya (bordering Ayano-Maysky administrative district westwards), but the character of changes remains unclear. The climate parameters in the nearest Nelkan Village (314 m a.s.l.) that is behind the main axis of Dzhugdzhur Range westwards (https://en.climate-data.org/asia/russian-federation/khabarovsk-krai/nelkan-296379/) has only 414 mm of precipitation per year (i.e. less than half that of Ayan), the mean annual temperature –6.7°C, with the warmest month being July, 14.4°C and the coldest one being January, –30.4°C. Therefore, the Nelkan Village climate is much more continental than that observed in the Ayan Settlement.

The vegetation in the study area is difficult to describe in distinct definitions of altitudinal belts. In fact, the dominant vegetation is represented by deeply interpenetrating complexes, such as forests composed of *Picea ajanensis* or *Larix cajanderi *Mayr, crooked forests (krummholz) composed of *Pinus pumila* (Pall.) Regel. and *Alnus fruticosa* Rupr. and mountain tundra and tundra-like communities. Indeed, the area just near to the seacoast provides a variation of communities from tundra (or, at least, almost indistinguishable analogue) at the slopes with severe wind conditions to true boreal dark-coniferous forests in the narrow valleys protected from the dominant winds (Fig. 2a). At elevations 300–400 m a.s.l., all abovementioned vegetation types occupied almost equal proportions. Azonal complexes are represented by vegetation of ice glades, rocky fields (gravelly barrens), swamps and floodplains. Rocky fields (kurum), depending on the topography, may be observed from the seashore, although they begin to dominate at elevations exceeding 500–700 m a.s.l. (Fig. 2b, c). The ice glades are occupied by willow-grass communities, sometimes with some mosses dominating in the cover, but commonly entirely liverworts free. Swamps are very local; we observed a true swamp (with peat deposits production) only once (although, it was quite peculiar due to sluggishly-flowing waters above a limestone sub-base) (Fig. 2d). Therefore, the use of terms such as boreal, subalpine and alpine belt bears only a conditional sense and reflects rather the nearest environment where the specimen was collected.

With the aforementioned reservations, the lower altitudes are dominated by spruce forests formed by *Picea ajanensis* that are usually mixed with *Betula lanata* in various proportions. In more drained areas and commonly further from the seacoast, closed to open *Larix cajanderii *stands (Fig. 3a) with common (almost inevitable) *Pinus pumila* (Pall.) Regel clumps in the understorey are widely developed. The riversides in wide floodplains of some rivers are occupied by communities composed of *Populus suaveolens* Fisch., *Chosenia arbutifolia* (Pall.) A.K. Skvortsov and *Salix udensis* Trautv. & C.A. Mey. with tall grass cover. Some shrubs, such as *Rosa* *acicularis *Lindl., *Sorbus sambucifolia *(Cham. & Schltdl.) M. Roem.**(the latter also often penetrates to the virgin birch forests), are almost confined to these communities. Pure *Betula lanata* (Regel) V.N. Vassil. stands are quite rare and are usually confined to the gentle slopes moistened by percolating waters near the base of hills and high slopes (Fig. 3b). The ground cover in birch forest is usually grassy, more rarely shrubby.

**Figure 4.**
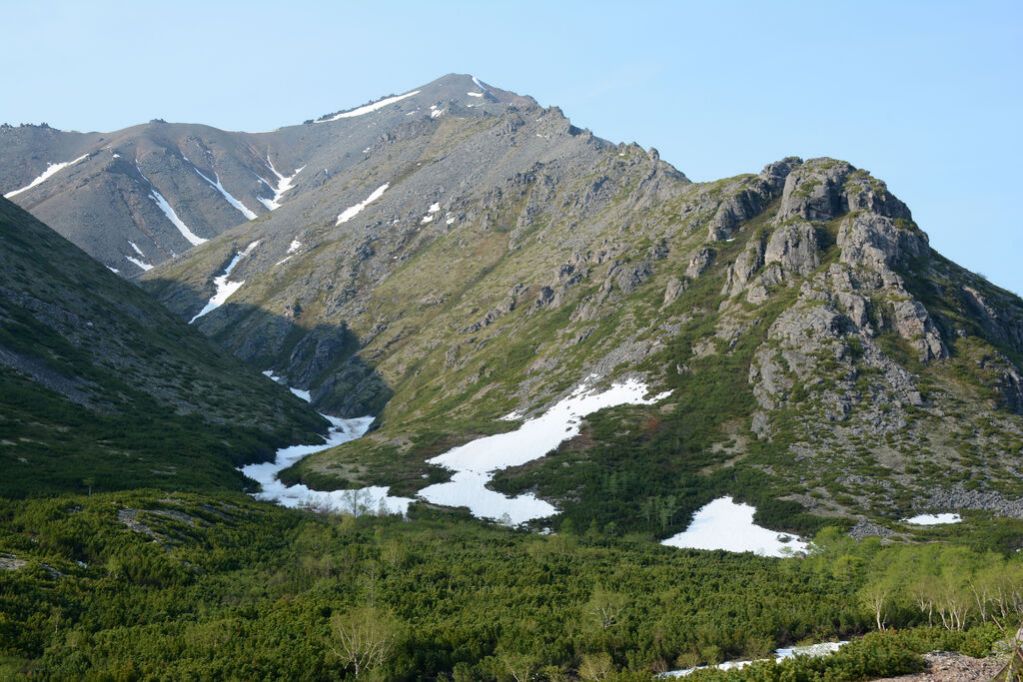
*Pinus pumila* (Pall.) Regel thickets (900 m a.s.l.) turning into scattered *Pinus pumila* clumps above, intermingled with a rocky field in the foreground; rock fields and spots of tundra (stream valley in axial part of Dzhugdzhur Range) are in the background. Photo by K.G. Klimova.

*Pinus pumila* – the main dominant plant in the area investigated (by total coverage) – forms well-defined stands (Figs 3b, 4). It almost does not penetrate to the spruce forests, but is widespread as an understorey in *Larix* forests and commonly so (although with much lesser regularity) in *Betula lanata* scattered forests. The virgin stands of *Pinus pumila* start at elevations of 100–200 m a.s.l. and usually remain widespread on the ridgelines of the spurs in the middle elevation, near 500–700 m a.s.l. Above these elevations, as far as to elevations exceeding 1000 m a.s.l., *Pinus pumila* clumps become more and more scattered, plants become smaller and more certainly better developed in the hollows protected from the wind, with thicker snow cover. Communities of *Duschekia fruticosa* – another dominant of crooked forests in the Hemiarctic Pacific Asia – are much rarer and likely confined to those places close to the surface or open percolating waters, as well as to the slopes of narrow mountain valleys where a lot of snow is deposited in winter. In addition, shrubby *Betula fruticosa* clumps sporadically occur near the axial part of Dzhugdzhur Range and form small-sized communities composed of dwarf modifications of the species and show a transition to the true mountain dwarf shrub tundra. Besides communities where *Betula fruticosa* is a dominant, that species commonly occurs in larch forests, where it reaches a height of 1 m or slightly more. In humid sites near the seacoast, where larch forests are replaced by mixed *Picea ajanensis* – *Betula lanata* forests, a continuous morphological transitional series of hybrids between *B. fruticosa* and *B. lanata* are observed, these hybrids commonly having a height between 1.5 and 3 m.

**Figure 6.**
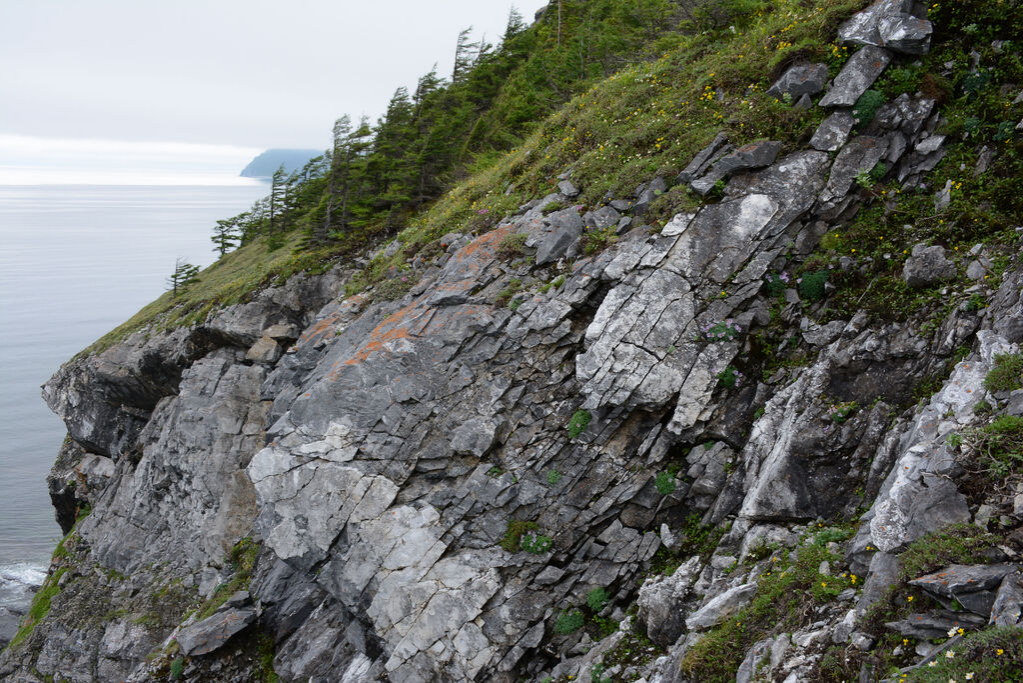
Steep NNE-facing slope to sea with limestone outcrops surrounded by tundra-like communities and ‘flag-formed’ trees of *Larix cajanderi* Mayr (34 m a.s.l.) (northwest of Ayan Settlement). Photo by K.G. Klimova.

*Pinus pumila* communities are not so much interrupted by mountain tundra (we rarely observed sizable mountain tundra communities), but they are mostly disconnected by rock fields (kurums), both on steep and gently sloping surfaces (Fig. 2b, c). In general, the water deficit (due to the absence of a closely lying waterproof horizon - in other words, when all the moisture after snowmelt or rains almost immediately seeps into the substrate), is the main reason for the absence of closed vegetation at heights exceeding 500–1000 m a.s.l. Mountain tundras are represented by scattered ‘spots’ of 5–20 m in length, less often longer and are located at the places of (or near) percolating water openings. The general organisation of alpine communities is usually exhausted by the following scheme: *Sphagnum lenense* tufts form the central patch, then they are surrounded by the perimeter (sometimes quite wide strip) of lichen-dwarf shrub (sometimes including dwarfy *Pinus pumila*) communities and then grade into rocky fields (Fig. 5a). Pure lichen tundra communities (Fig. 5b) are distributed somewhat wider than moss and moss-shrub communities, although also are not common. The special conditions for the development of wet dwarf shrub-moss tundra are observed at the base of slopes at heights exceeding 1100 m a.s.l., in places of firns (dense snow strata lying sometimes over summer) distribution and/or groundwater openings. The severe wind regime on the seacoast, especially in the saddles between the hill-shaped uplifts, leads to the local distribution of tundra-like communities even at altitudes less than 50 m a.s.l. (Figs 5c, d, 6). At a distance from the seacoast, such communities are found everywhere above 300 m a.s.l. and are confined to the most well-drained axial parts of small spurs with clearly blown snow in the winter.

In general, the vegetation of the study area can be identified as subarctic montane. The spruce forests do not dominate even in the lower altitudes and, as a rule, have a great admixture of hem-iarctic *Betula lanata*. *Larix* forests have an understorey of *Pinus pumila* and commonly do not have closed canopy. The larger areas are covered by the ‘subalpine’ shrub *Pinus pumila* that occurs as understorey or small-sized clumps from lower elevations starting almost from the seacoast and then stretching to the elevations slightly exceeding 1000 m a.s.l., as dwarf and strongly disintegrated patches. The subarctic nature of the flora is also determined by the climate characteristics briefly described above.

#### Data analyses

**Figure 7.**
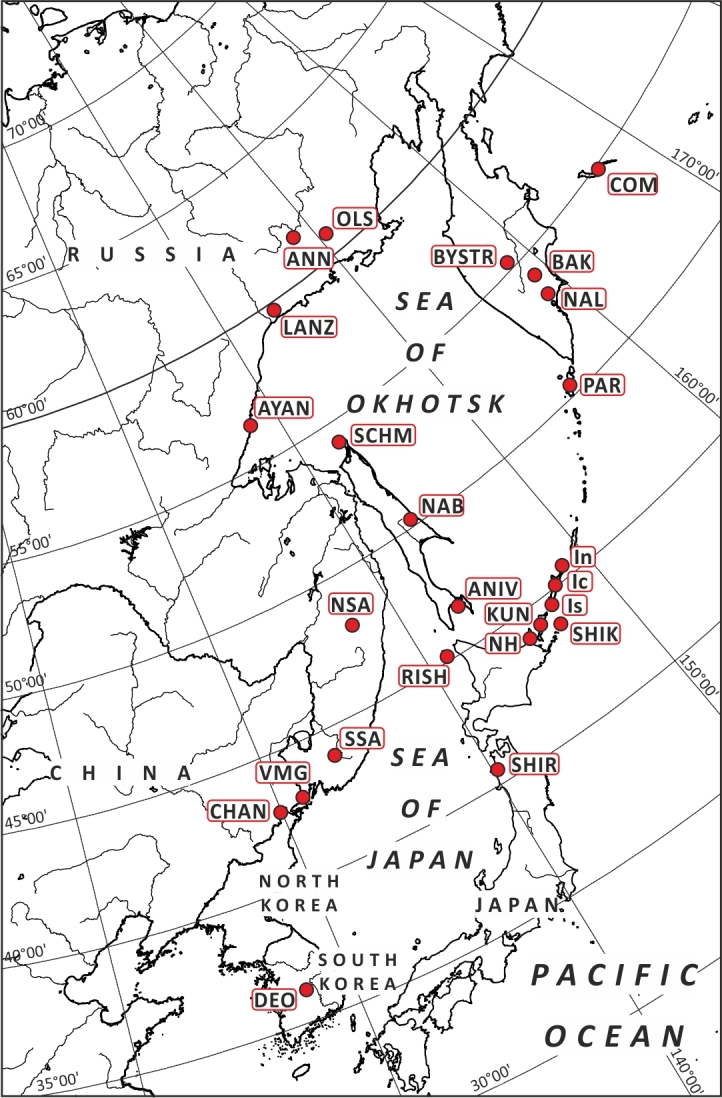
The floras involved in the comparative analysis (abbreviation are explained in Table 2).

To evaluate the position of the studied flora within Northeast Asian floras, we have used the Detrended Correspondence Analysis (DCA), based on the matrix that is largely the same as that used in the identification of the position of bryophyte flora of northern Iturup Island (Bakalin et al. 2019). Besides Ayan flora, the data on East-Manchurian Mountains, united floras of northern part and the southern part (instead of flora of southern Sikhote-Alin) of Sikhote-Alin Mts. were added too. The comparison involves 25 floras (Table 2, Fig. 7) and is based on a matrix (Suppl. material 1) where each species was marked as 1 (presence) or 0 (absence). These data were tested using multivariate analysis (using Past ver. 4.03c (Hammer et al. 2001)). The hierarchical clustering was based on Ward’s method (Ward 1963), Euclidean distance to check the results shown by DCA. DCA was visualised in three-dimensional grid graph, with the third dimension given by colour gradient.

## Results

### Annotated Checklist of Liverworts

One hundred and seventeen species and one subspecies were revealed in the flora of Ayan surroundings, as was defined in the present study. All identification data were input into the electronic herbarium database of the Botanical Garden-Institute FEB RAS (http://botsad.ru/en/herbarium/). All taxa are listed below in alphabetical order. Nomenclature follows Söderström et al. (2016), with the exception for: 1) Solenostomataceae, where the narrow genus concept was adopted; 2) acceptance of *Pseudolophozia* distinct from *Barbilophozia*; 3) new conception of *Schistochilopsis* genus according to Bakalin et al. (2020b) and 4) the narrow species concept in *Blepharostoma* (the last following Bakalin et al. (2020a)). As only a few specimens of *Blepharostoma *species (only in pure mats) were checked by DNA analysis, clear taxonomical status of the vast majority of *Blepharostoma *species found as accompanying taxa (sometimes in small amounts) to other liverworts is not known definitely. In one case (stressed in the checklist), the name *Blepharostoma *sp. from Kh-58-4-19 means new cryptic taxon discussed in Bakalin et al. (2020a). *Cladopodiella* is accepted in the old sense, as the genus is different from *Odontoschisma*. Each taxon is annotated with data on: 1) numbers of collecting localities in accordance with Table 1 and Fig. 1, numbers are given in bold font; 2) altitudinal diapason in the explored area, m above sea level; 3) description of habitat in area treated and accompanying taxa and 4) selected specimens examined field numbers. The subsections are divided by dash.

***Anastrophyllum michauxii*** (F. Weber) H. Buch – **23** – 118 m – Moist decaying wood in *Picea* *ajanensis* dominated forest with *Betula lanata* slight admixture, in the stream valley. With *Mylia verrucosa*. Kh-55-16-19.

***Aneura pinguis*** (L.) Dumort. – **16**, **25**, **26** – 22–105 m – Open moist to wet places including humus in the trail in open *Larix* forest, hummocks in the meadow-like windy community near seacoast with low grasses and shrubs and hollows in eutrophic hypnaceous swamp developed over limestone, with sluggishly-flowing waters. In pure mats or with *Mesoptychia rutheana*. Kh-48-4-19, Kh-58-9-19, Kh-59-2-19, Kh-59-3-19.

***Anthelia juratzkana*** (Limpr.) Trevis. – **6**, **7**, **9**, **10**, **12**, **15**, **18** – 129–1484 m – Open or rarely partly shaded places in cliffy areas in the forests and alpine belt, most commonly on moist cliffs and rocks, including those near streams, rare in their crevices or humus along watercourses in a variety of communities, such as scattered *Betula lanata* forest along riverside, *Picea ajanensis* dominated forest in narrow valley, mosaic of *Duschekia* and *Pinus pumila* clumps and mountain tundra vegetation on steep N-facing slopes, moist mossy tundra near stream and alpine vegetation with many rocky outcrops and gravelly barrens on steep N-facing slope to stream. In pure mats or with *Blepharostoma *sp.,* Cephalozia bicuspidata*,* Cladopodiella francisci*,* Cryptocolea imbricata*,* Diplophyllum taxifolium*,* Gymnocolea inflata*,* Marsupella boeckii*,* Marsupella emarginata*,* Nardia geoscyphus*,* Pleurocladula albescens*,* Prasanthus suecicus*,* Protochilopsis grandiretis*,* Pseudolophozia debiliformis*,* Pseudolophozia sudetica*,* Pseudotritomaria heterophylla*,* Radula prolifera*,* Scapania *sp.,* Solenostoma obscurum*,* Sphenolobus minutus* and* Tetralophozia setiformis*. Kh-38-9-19, Kh-39-10-19, Kh-41-6-19, Kh-42-3-19, Kh-44-1-19, Kh-47-26-19, Kh-50-53-19, Kh-56-21-19.

***Barbilophozia barbata*** (Schreb.) Loeske – **2**, **3** – 141–294 m – Mesic cliffs and boulders in *Larix* forest with some clumps of *Pinus pumila* in understorey and upper reaches of subalpine belt near the top of the hill covered mostly with the mosaic of alpine and subalpine scattered vegetation. In pure mats or with *Metzgeria pubescens* and *Scapania microdonta*. Kh-34-2-19, Kh-35-11-19, Kh-35-14-19.

***Barbilophozia hatcheri*** (A. Evans) Loeske – **19** – 780 m – Open mesic cliff crevice at the mountain ridge just above crooked forest line. With *Scapania sphaerifera*,* Sphenolobus saxicola* and* Tetralophozia setiformis*. Kh-51-9-19.

***Bazzania denudata*** (Torr. ex Gottsche, Lindenb. & Nees) Trevis. – **17**, **18** – 82–154 m – Open to partly shaded moist cliffs and their crevices in *Betula lanata*, sometimes scattered forests along riversides. In pure mats or with *Bazzania tricrenata*,* Blepharostoma *sp.,* Blepharostoma trichophyllum*,* Diplophyllum taxifolium*,* Douinia plicata*,* Lepidozia reptans* and* Metacalypogeia cordifolia*. Kh-49-5-19, Kh-50-6-19, Kh-50-44-19, Kh-56-16-19.

***Bazzania tricrenata*** (Wahlenb.) Trevis. – **18** – 129–154 m – Open moist to mesic large riverside N-facing cliffs, with scattered *Betula lanata* forest. In pure mats or with *Bazzania denudata*,* Calycularia laxa*,* Cephaloziella *sp.,* Diplophyllum taxifolium*,* Douinia plicata*,* Herbertus *aff.* buchii*,* Marsupella emarginata*,* Metacalypogeia cordifolia*,* Plagiochila porelloides*,* Solenostoma rossicum*,* Sphenolobus minutus*,* Trilophozia quinquedentata* and* Tritomaria exsecta*. Kh-50-23-19, Kh-50-39-19, Kh-50-48-19, Kh-50-56-19, Kh-56-14-19.

***Blepharostoma primum*** Vilnet et Bakalin – **15** – 143 m – Open moist cliff near stream in *Picea ajanensis* dominating forest in the valley. In pure mats. Kh-47-28-19.

***Blepharostoma trichophyllum*** (L.) Dumort. s. str. – **14**, **18** – 54–154 m – Partly shaded moist cliffs and open wet humus near the stream in scattered pure *Betula lanata* and mixed *Picea ajanensis*-*Betula lanata* forests in the valleys. In pure mats. Kh-46-12-19, Kh-50-17-19.

***Blepharostoma neglectum *"*neglecta*"****Vilnet et Bakalin – **14** – 54 m – Moist fallen decaying tree branch in *Picea ajanensis*-*Betula lanata* forest in the valley. In pure mats. Kh-46-3-19.

***Blepharostoma*** sp. (indefinite hybrid taxon with *B. trichophyllum *as one parent, cf. Bakalin et al. 2020) – **25** – 22 m – Partly shaded moist humus developed over limestone on the trail in open *Larix* forest in small stream valley. With *Eocalypogeia schusteriana*, *Mesoptychia gillmanii* and *Scapania gymnostomophila*. Kh-58-4-19.

***Calycularia laxa*** Lindb. & Arnell – **6**, **7**, **11**, **15**, **18** – 129–1261 m – Partly shaded crevices in the cliffs and between boulders, also those filled with fine soil. Boreal forests including pure stands by *Picea ajanensis*, *Betula lanata* and their mixed variants, large riverside N-facing cliffs with scattered *Betula lanata*; mosaic of *Duschekia* and *Pinus pumila* clumps and mountain tundra vegetation on steep slopes; dwarf shrub-lichen tundra with gravelly barrens on steep N-facing slopes. In pure mats or with *Bazzania tricrenata*,* Diplophyllum taxifolium*,* Lophozia *sp.,* Marsupella emarginata*,* Sphenolobus minutus*,* Sphenolobus saxicola* and* Trilophozia quinquedentata*. Kh-38-12-19, Kh-39-5-19, Kh-43-4-19, Kh-47-15-19, Kh-50-35-19, Kh-56-1-19.

***Calypogeia integristipula*** Steph. – **1**, **15** – 11–143 m – Partly shaded moist humus on slopes, decaying wood and cliffs near stream in *Picea ajanensis* dominating forest in narrow valley. With *Blepharostoma *sp., *Cephalozia bicuspidata*,* Douinia plicata*,* Fuscocephaloziopsis lunulifolia*,* Lepidozia reptans*,* Lophozia *sp.,* Scapania parvifolia*,* Schistochilopsis incisa* and* Sphenolobus minutus*. Kh-33-11-19, Kh-47-5-19, Kh-47-14-19, Kh-47-16-19, Kh-47-18-19.

***Calypogeia muelleriana*** (Schiffn.) Müll. Frib. – **11** – 1148 m – Over wet *Sphagnum* mat in alpine belt with scattered vegetation over percolating water. With *Lophozia* sp., *Scapania parvifolia* and* Sphenolobus minutus*. Kh-43-11-19.

***Calypogeia neogaea*** (R.M. Schust.) Bakalin – **6** – 904 m – Over moist *Sphagnum* mat on the slope in mosaic of *Duschekia* and *Pinus pumila* clumps and mountain tundra vegetation. With *Cephalozia* sp. and *Neoorthocaulis binsteadii*. Kh-38-7-19.

***Calypogeia suecica*** (Arnell & J. Perss.) Müll. Frib. – **23** – 118 m – Moist decaying wood in *Picea* forest with scattered *Betula lanata* admixture in the valley. With *Cephalozia bicuspidata*,* Lophozia guttulata* and* Mylia verrucosa*. Kh-55-10b-19.

***Cephalozia bicuspidata*** (L.) Dumort. – **1**, **7**, **9**, **11**, **14**, **15**, **23**, **26** – 11–1484 m – Moist humus on the trails, open wet peat, clayish soil on steep slopes, rarer in dense moist mossy hummocks. Mixed *Picea ajanensis*-*Betula lanata* forests, through subalpine belt where near *Duschekia* and *Pinus pumila* clumps to alpine belt with scattered vegetation, once in eutrophic hypnaceous swamp developed over limestone, with sluggishly-flowing water above. In pure mats or with *Anthelia juratzkana*,* Calypogeia integristipula*,* Calypogeia suecica*,* Diplophyllum taxifolium*,* Lophozia guttulata*,* Lophozia silvicoloides*,* Lophozia *sp.,* Marsupella emarginata*,* Mylia verrucosa*,* Nardia geoscyphus*,* Prasanthus suecicus*,* Scapania irrigua*,* Scapania parvifolia*,* Schistochilopsis incisa*,* Solenostoma obscurum*,* Sphenolobus minutus* and* Trilophozia quinquedentata*. Kh-33-7-19, Kh-39-9-19, Kh-41-6-19, Kh-43-6-19, Kh-46-1-19, Kh-47-22-19, Kh-55-10-19, Kh-59-1-19.

***Cephaloziella divaricata*** (Sm.) Schiffn. – **17**, **19** – 82–780 m – Open moist cliffs and their crevices in large riverside S-facing cliffs in scattered *Picea ajanensis* forest and cliffs on ridgeline of small spur in crooked forest belt. With *Mannia fragrans*,* Mannia fragrans *var.* fragrans*,* Mannia sibirica* and* Riccia sorocarpa*. Kh-49-11-19, Kh-49-21-19, Kh-49-22-19, Kh-49-22a-19, Kh-51-2-19.

***Cephaloziella varians*** (Gottsche) Steph. – **9**, **11** – 1148–1484 m – Open mesic sandy soil on slope in alpine belt with sparse vegetation. With *Cryptocolea imbricata*,* Fuscocephaloziopsis pleniceps*,* Isopaches bicrenatus*,* Riccardia palmata*,* Scapania *sp. and* Protochilopsis grandiretis*. Kh-41-9-19, Kh-43-1-19.

***Cladopodiella francisci*** (Hook.) Jørg. – **12** – 1109 m – Over humus on wet mossy bank of the stream in moist mossy tundra. With *Anthelia juratzkana*,* Pseudolophozia sudetica*. Kh-44-4-19.

***Conocephalum salebrosum*** Szweyk., Buczk. & Odrzyk. – **18**, **25** – 22–154 m – Open to partly shaded moist humus covering rocks in large N-facing conglomerate cliffs with scattered *Betula lanata* along riverside and in open *Larix* forest in small stream valley with many limestone boulders in the streambed. In pure mats or with *Plagiochila porelloides*. Kh-56-5-19, Kh-58-8-19.

***Crossocalyx hellerianus*** (Nees ex Lindenb.) Meyl. – **3**, **23** – 118–294 m – Partly shaded moist decaying decorticated fallen tree trunk in *Larix* forest with some clumps of *Pinus pumila* in understorey. With *Lepidozia reptans*,* Lophozia *sp.,* Mylia verrucosa* and* Ptilidium pulcherrimum*. Kh-35-8-19, Kh-35-9-19, Kh-55-8-19.

***Cryptocolea imbricata*** R.M. Schust. – **9**, **10** – 1478–1484 m – Open wet humus along stream and moist sandy soil on slope, only in the areas of distribution of rocks of basic reaction in alpine belt with scattered vegetation. With *Anthelia juratzkana*,* Cephaloziella varians*,* Fuscocephaloziopsis pleniceps*,* Pseudotritomaria heterophylla*,* Radula prolifera*,* Riccardia palmata*,* Scapania *sp. and* Protochilopsis grandiretis*. Kh-41-3-19, Kh-42-3-19.

***Diplophyllum sibiricum*** Bakalin et Vilnet – **17** – 82 m – Open moist cliff in large riverside S-facing cliffs with scattered *Betula lanata*. With *Diplophyllum taxifolium*,* Marsupella emarginata*,* Scapania mucronata* and* Schistochilopsis* sp. Kh-49-16-19.

***Diplophyllum taxifolium*** (Wahlenb.) Dumort. – **2**, **7**, **15**, **17**, **18** – 82–1261 m – Open to partly shaded moist cliffs and boulders in *Picea ajanensis* forests, large riverside cliffs with scattered *Betula lanata* and alpine vegetation with many rocky outcrops and gravelly barrens on steep N-facing slope. In pure mats or with *Anthelia juratzkana*,* Bazzania denudata*,* Bazzania tricrenata*,* Calycularia laxa*,* Cephalozia bicuspidata*,* Diplophyllum sibiricum*,* Douinia plicata*,* Frullania subarctica*,* Gymnomitrion corallioides*,* Lepidozia reptans*,* Marsupella boeckii*,* Marsupella emarginata*,* Metacalypogeia cordifolia*,* Metzgeria pubescens*,* Nardia geoscyphus*,* Plagiochila porelloides*,* Pleurocladula albescens*,* Pseudolophozia sudetica*,* Scapania crassiretis*,* Scapania mucronata*,* Schistochilopsis incisa*,* Schistochilopsis *sp.,* Solenostoma obscurum*,* Sphenolobus minutus* and* Trilophozia quinquedentata.* Kh-34-3-19, Kh-39-12-19, Kh-47-8-19, Kh-49-18-19, Kh-50-42-19.

***Douinia plicata*** (Lindb.) Konstant. et Vilnet – **1**, **14**, **15**, **17**, **18**, **23** – 11–154 m – Partly shaded moist decaying wood, moist cliffs, rarely clayish soil on steep slopes; from various coniferous, mixed and *Betula lanata* forests to subalpine belt with many *Duschekia* and *Pinus pumila* clumps. In pure mats or with *Bazzania denudata*,* Bazzania tricrenata*,* Blepharostoma trichophyllum*,* Calypogeia integristipula*,* Diplophyllum taxifolium*,* Fuscocephaloziopsis lunulifolia*,* Lepidozia reptans*,* Lophozia *sp.,* Marsupella emarginata*,* Metacalypogeia cordifolia*,* Metzgeria pubescens*,* Schistochilopsis incisa* and* Sphenolobus minutus*. Kh-33-5-19, Kh-46-17-19, Kh-47-14-19, Kh-49-24-19, Kh-50-23-19, Kh-55-19-19, Kh-56-17-19.

***Eocalypogeia schusteriana*** (S. Hatt. & Mizut.) R.M. Schust. – **18**, **24** – 34–154 m – Open to, rarely, partly shaded crevices in cliffs of basic to neutral reaction (mostly metamorphic rocks, rarely pure limestone), in windy tundra-like communities near seacoast and scattered *Betula lanata* stands along sea coast and riverside. With *Blepharostoma sp.*,* Jungermannia afoninae*,* Mesoptychia badensis*,* Mesoptychia gillmanii*,* Preissia quadrata* and* Scapania gymnostomophila*. Kh-50-16-19, Kh-50-34-19, Kh-57-8-19, Kh-57-11-19, Kh-57-19-19, Kh-57-23-19.

***Eremonotus myriocarpus*** (Carrington) Pearson – **18** – 129–154 m – Open moist cliff crevices in large N-facing conglomerate cliffs with scattered *Betula lanata* along riverside. With *Sauteria alpina* and* Solenostoma obscurum*. Kh-56-10a-19, Kh-56-12-19.

***Frullania bolanderi*** Austin – **4**, **15** – 143–377 m – Partly shaded *Betula lanata* and *Salix udensis* trunks in valley forests near streams and rivers. In pure mats. Kh-36-11-19, Kh-47-12-19, Kh-47-13-19.

***Frullania davurica*** Hampe – **18**, **19** – 129–780 m – Open mesic cliffs on riverside with scattered *Betula lanata* and ridgeline (crooked forest belt) of small ridge with many rocky outcrops, gravelly barrens and *Pinus pumila* clumps. In pure mats or with *Frullania ignatovii*,* Frullania subarctica* and* Trilophozia quinquedentata*. Kh-50-27-19, Kh-50-28-19, Kh-51-8-19.

***Frullania ignatovii*** Sofronova, Mamontov & Potemkin – **18** – 129–154 m – Open mesic cliffs in large riverside N-facing cliffs with scattered *Betula lanata*. In pure mats or with *Frullania davurica*. Kh-50-26-19, Kh-50-29-19.

***Frullania subarctica*** Vilnet, Borovich. et Bakalin – **18** – 129–154 m – Open moist cliffs in large riverside N-facing cliffs with scattered *Betula lanata*. In pure mats or with *Diplophyllum taxifolium*,* Frullania davurica*,* Frullania ignatovii*,* Gymnomitrion corallioides*,* Herbertus *aff.* buchii*,* Plagiochila porelloides* and* Scapania crassiretis*. Kh-50-43a-19, Kh-50-47-19, Kh-50-55-19, Kh-56-26-19.

***Fuscocephaloziopsis leucantha*** (Spruce) Váňa et L.Söderstr. – **23** – 118 m – Moist decaying wood in *Picea* forest with scattered *Betula lanata* in the valley. With *Lophozia guttulata*. Kh-55-10a-19.

***Fuscocephaloziopsis lunulifolia*** (Dumort.) Váňa et L.Söderstr. – **15**, **23** – 118–143 m – Moist decaying wood, moist humus on trails and humus covering cliffs in *Picea* forest also that with the admixture of *Betula lanata*. With *Blepharostoma* sp., *Calypogeia integristipula*,* Douinia plicata*,* Lepidozia reptans*,* Lophozia *sp.,* Mylia verrucosa*,* Schistochilopsis incisa* and* Schistochilopsis incisa*. Kh-47-2-19, Kh-47-14a-19, Kh-55-3-19.

***Fuscocephaloziopsis pachycaulis*** (R.M.Schust.) Váňa et L.Söderstr. – **15** – 118 m – Open moist cliff near stream in *Picea ajanensis* dominating forest in narrow valley. In pure mats. Kh-47-23-19.

***Fuscocephaloziopsis pleniceps*** (Austin) Váňa & L. Söderstr. – **9**, **12** – 1109–1484 m – Over humus on wet mossy bank of the stream in moist mossy tundra. With *Cephaloziella varians*,* Cryptocolea imbricata*,* Mylia anomala*,* Riccardia palmata*,* Scapania *sp. and* Protochilopsis grandiretis*. Kh-41-9-19, Kh-44-2-19, Kh-44-8-19.

***Gymnocolea inflata*** (Huds.) Dumort. – **6**, **12** – 904–1109 m – Mossy banks and open wet cliffs near streams in subalpine belt with mosaic of *Duschekia* and *Pinus pumila* clumps and mossy tundra. With *Anthelia juratzkana*,* Pleurocladula albescens*,* Protochilopsis grandiretis*,* Schljakovia kunzeana* and* Tetralophozia setiformis*. Kh-38-10-19, Kh-44-5-19, Kh-44-13-19.

***Gymnomitrion commutatum*** (Limpr.) Schiffn. – **6**, **8**, **9**, **19** – 780–1484 m – Mesic to moist sandy soil and humus on steep slopes and solifluction spots, rarely open moist cliffs, in subalpine and alpine belts. In pure mats and with *Gymnomitrion concinnatum*. Kh-38-4-19, Kh-40-9-19, Kh-41-1-19, Kh-51-4-19.

***Gymnomitrion concinnatum*** (Lightf.) Corda – **7**, **8** – 1261–1328 m – Open moist cliffs in alpine belt with scattered vegetation and many rocky outcrops and gravelly barrens on steep N-facing slope. With *Gymnomitrion commutatum*,* Gymnomitrion corallioides* and* Pseudolophozia sudetica*. Kh-39-3-19, Kh-40-4-19.

***Gymnomitrion*** ***corallioides*** Nees – **2**, **6**, **7**, **8**, **18**, **19** – 129–1328 m – Moist to mesic cliffs and their crevices, rarely dense sandy soil on slopes; in large riverside N-facing cliffs with scattered *Betula lanata*; subalpine belt with mosaic of *Duschekia* and *Pinus pumila* clumps and mountain tundra vegetation on steep N-facing slope and alpine belt with scattered vegetation with many rocky outcrops and gravelly barrens moistened by neutral to basic reaction percolating water on steep N-facing slope. In pure mats and with *Diplophyllum taxifolium*,* Frullania subarctica*,* Gymnomitrion concinnatum*,* Pseudolophozia sudetica*,* Scapania crassiretis* and* Sphenolobus minutus*. Kh-34-3-19, Kh-38-3-19, Kh-39-3-19, Kh-40-3-19, Kh-50-52-19, Kh-51-10-19.

***Herbertus ***aff. ***buchii*** Juslen – **18**, **22** – 119–154 m – Kh-50-1-19, Kh-50-25-19, Kh-50-43-19, Kh-54-4-19, Kh-56-27-19, Kh-56-28-19. Open moist cliffs along riverside with scattered *Betula lanata*, rarely moist boulders in waterfall canyon in subalpine belt. In pure mats or with *Bazzania tricrenata*,* Cephaloziella *sp.,* Frullania subarctica*,* Marsupella emarginata*,* Metacalypogeia cordifolia*,* Plagiochila porelloides*,* Scapania crassiretis*,* Solenostoma rossicum* and* Sphenolobus minutus*.

***Isopaches bicrenatus*** (Schmidel) H. Buch – **10**, **11** – 1148–1478 m – Open mesic clayish soil on slope with scattered alpine vegetation, many rocky outcrops and gravelly barrens moistened by neutral to basic reaction percolating water on steep N-facing slope. With *Cephaloziella varians*. Kh-42-1-19, Kh-43-1-19.

***Jungermannia afoninae*** Mamontov, Konstant. & Vilnet – **17**, **21**, **24** – 34–235 m – Open to partly shaded moist cliff crevices and niches in lower elevations: large riverside cliffs with scattered *Betula lanata*, windy tundra-like communities developed over limestone and deep canyons near waterfalls. In pure mats and with *Eocalypogeia schusteriana*,* Mesoptychia badensis*,* Preissia quadrata* and* Scapania gymnostomophila*. Kh-49-1-19, Kh-49-14-19, Kh-53-2-19, Kh-57-1-19, Kh-57-12-19, Kh-57-13-19.

***Jungermannia pumila*** With. – **21** – 235 m – Open wet boulder near stream in deep canyon with small waterfall, in subalpine belt with scattered *Pinus pumila* clumps in surroundings. With *Scapania irrigua*. Kh-53-7-19.

***Lejeunea alaskana*** (R.M. Schust. & Steere) Inoue & Steere – **18** – 129–154 m – Open moist cliff in large N-facing conglomerate rocky outcrops with scattered *Betula lanata* along riverside. In pure mats. Kh-56-24-19.

***Lepidozia reptans*** (L.) Dumort. – **15**, **17**, **21**, **24** – 34–235 m – Moist fallen decaying decorticated tree trunks, decaying stumps, moist cliffs and their crevices, everywhere in part shade; in forest belt, including *Picea ajanensis* forest with scattered *Betula lanata* trees, *Picea ajanensis-Betula lanata* forest in the valley with some *Salix udensis* near stream, *Larix* forest in narrow valley and scattered *Betula lanata* stand in large riverside cliffs. With *Bazzania denudata*,* Blepharostoma *sp.,* Blepharostoma trichophyllum*,* Calypogeia integristipula*,* Crossocalyx hellerianus*,* Diplophyllum taxifolium*,* Douinia plicata*,* Fuscocephaloziopsis lunulifolia*,* Lophozia guttulata*,* Lophozia *sp.,* Metacalypogeia cordifolia*,* Mylia verrucosa*,* Schistochilopsis incisa*,* Sphenolobus minutus* and* Tritomaria exsecta*. Kh-36-3-19, Kh-46-7-19, Kh-47-16-19, Kh-49-6-19, Kh-50-7-19, Kh-55-15-19, Kh-55-18-19.

***Lophocolea heterophylla*** (Schrad.) Dumort. – **4** – 377 m – Partly shaded moist fallen decaying decorticated tree trunk in wide floodplain with solitary *Populus suavealons* and *Salix udensis* trees near streambed. With *Blepharostoma* sp. Kh-36-9-19.

***Lophocolea minor*** Nees – **1**, **4**, **25** – 11–377 m – Partly shaded moist humus along trail, boulders on slopes, fallen decaying tree trunks; in open *Larix* forests, subalpine belt with many *Duschekia* and *Pinus* *pumila* clumps, floodplain *Populus suavealons* and *Salix udensis* forest near streambed. In pure mats and with *Mesoptychia* aff. *heterocolpos*. Kh-33-3a-19, Kh-36-10-19, Kh-58-3-19.

***Lophozia guttulata*** (Lindb. & Arnell) A. Evans – **14**, **15**, **23** – 54–143 m – Partly shaded moist decaying wood, rarely humus covered boulders in *Picea ajanensis* and *Betula lanata* pure stands and their mixtures. With *Calypogeia suecica*,* Cephalozia bicuspidata*,* Fuscocephaloziopsis leucantha*,* Lepidozia reptans*,* Lophozia silvicola* and* Mylia verrucosa*. Kh-46-8-19, Kh-47-4-19, Kh-55-10-19, Kh-55-14-19.

***Lophozia lantratoviae*** Bakalin – **18**, **20** – 129–200 m – Open and partly shaded cliffs and their crevices along streams and riversides in scattered *Betula lanata* forest and floodplain *Salix udensis-Chosenia arbutifolia-Populus suaveolens *stand*.* With* Blepharostoma *sp.,* Mesoptychia heterocolpos*,* Scapania gymnostomophila*,* Scapania rufidula* and* Trilophozia quinquedentata*,* Tritomaria scitula*. Kh-50-32-19, Kh-52-1a-19, Kh-56-13-19, Kh-56-19-19.

***Lophozia silvicola*** H. Buch – **3**, **15**, **18** – 129–294 m – Open to partly shaded moist humus (also that covering rocks) along streams and rivers in scattered *Betula lanata* forest and flood plain communities dominated by *Populus suavealons* and *Salix udensis*. In pure mats or with *Lophozia guttulata*. Kh-35-2-19, Kh-47-4-19, Kh-50-10-19.

***Lophozia silvicoloides*** N. Kitag. – **1**, **3** – 11–294 m – Partly shaded moist clayish soil on steep slope and decaying wood; in *Larix* forest in narrow valley and subalpine belt with clumps of *Duschekia* and *Pinus pumila*. With *Cephalozia bicuspidata* and* Sphenolobus minutus*. Kh-33-8-19, Kh-35-7-19.

***Lophozia ventricosa*** (Dicks.) Dumort. – **14**, **23** – 54–118 m – Moist decaying wood and partly shaded moist humus near stream in *Picea ajanensis–Betula lanata* forests, also those near streams with scattered *Salix udensis*. In pure mats or with *Mylia verrucosa*. Kh-46-15-19, Kh-55-7-19.

***Lophoziopsis excisa*** (Dicks.) Konstant. & Vilnet – **17** – 82 m – Open moist crevices in large riverside S-facing cliffs with scattered *Betula lanata*. In pure mats. Kh-49-9-19.

***Lophoziopsis longidens*** (Lindb.) Konstant. et Vilnet – **4** – 377 m – Partly shaded lying *Pinus pumila* branches in *Larix* forest with scattered *Pinus pumila* in understorey. In pure mats. Kh-36-4-19.

***Mannia androgyna*** (L.) Evans – **17** – 82 m – Partly shaded moist crevice in large riverside S-facing cliffs in forest belt. In pure mats. Kh-49-2-19.

***Mannia fragrans*** (Balb.) Frye & L. Clark – **17** – 82 m – Open moist crevice in large riverside S-facing cliffs in forest belt. In pure mats or with *Cephaloziella divaricata*. Kh-49-10-19, Kh-49-12-19

***Mannia pilosa*** (Hornem.) Frye & L. Clark – **18**, **24**, **27**, **28** – 5–154 m – Open moist cliff crevices partly filled with humus; in large riverside basic conglomerate S-facing cliffs surrounded by scattered *Betula lanata* and limestone outcrops surrounded by tundra-like community. In pure mats or with *Mesoptychia* sp., *Preissia quadrata* and* Scapania gymnostomophila*. Kh-50-4-19, Kh-50-5-19, Kh-50-31-19, Kh-57-4-19, Kh-57-5-19, Kh-60-3-19, Kh-61-2-19.

***Mannia*** ***sibirica*** (K. Müller) Frye & L. Clark – **17** – 82 m – Open moist crevice in large riverside S-facing cliffs with scattered *Betula lanata*. With *Cephaloziella divaricata*,* Riccia sorocarpa* and* Riccia bifurca*. Kh-49-3-19, Kh-49-13-19.

***Marchantia alpestris*** (Nees) Burgeff – **27** – 5 m – Partly shaded moist clay in deep niche in sedimentary N-facing rocky outcrops of basic reaction along seacoast. In pure mats. Kh-60-1-19.

***Marsupella boeckii*** (Austin) Lindb. ex Kaal. – **15** – 143 m – Open moist boulders and cliffs near stream in *Picea ajanensis* dominating forest in narrow valley. With *Anthelia juratzkana*,* Diplophyllum taxifolium*,* Nardia geoscyphus*,* Pleurocladula albescens*,* Pseudolophozia sudetica* and* Schistochilopsis incisa*. Kh-47-1-19, Kh-47-19-19, Kh-47-24-19.

***Marsupella emarginata*** (Ehrh.) Dumort. – **7**, **8**, **15**, **17**, **18**, **21** – 82–1328 m – Open to partly shaded moist cliffs near streams and rivers as well as in waterfall canyons, from *Picea ajanensis* forests to alpine belt. In pure mats or with *Anthelia juratzkana*,* Bazzania tricrenata*,* Blepharostoma *sp.,* Calycularia laxa*,* Cephalozia bicuspidata*,* Diplophyllum sibiricum*,* Diplophyllum taxifolium*,* Douinia plicata*,* Herbertus *aff.* buchii*,* Metacalypogeia cordifolia*,* Pleurocladula albescens*,* Pseudolophozia sudetica*,* Scapania mucronata*,* Schistochilopsis incisa*,* Schistochilopsis *sp.,* Sphenolobus minutus* and* Trilophozia quinquedentata*. Kh-39-6-19, Kh-39-11-19, Kh-40-5-19, Kh-40-7-19, Kh-47-25-19, Kh-49-17-19, Kh-50-24-19, Kh-50-40-19, Kh-50-41-19, Kh-53-1-19, Kh-56-18-19, Kh-56-25-19.

***Marsupella sprucei*** (Limpr.) Bernet – **6**, **8** – 904–1328 m – Open moist peaty and sandy soil on N-facing slopes in alpine and subalpine belts. In pure mats. Kh-38-1-19, Kh-40-1-19.

***Mesoptychia badensis*** (Gottsche ex Rabenh.) L. Söderstr. & Váňa – **24** – 34 m – Open moist limestone cliffs also those covered with thin layer of humus, surrounded by tundra-like community in low elevation near seacoast. In pure mats or with *Eocalypogeia schusteriana*,* Jungermannia afoninae* and* Scapania gymnostomophila*. Kh-57-6-19, Kh-57-14-19, Kh-57-15-19.

***Mesoptychia gillmanii*** (Austin) L. Söderstr. & Váňa – **24** – 34 m – Open moist clayish soil on steep slope with many limestone outcrops surrounded by tundra-like community near seacoast. With *Blepharostoma sp.*,* Eocalypogeia schusteriana*,* Plagiochila satoi* and* Preissia quadrata*. Kh-57-16-19, Kh-57-17-19.

***Mesoptychia heterocolpos*** (Thed.) L. Söderstr. & Váňa – **17**, **18**, **25** – 22–154 m – Open moist clayish soil along watercourse, moist cliff crevices and, rarely, partly shaded decaying wood in scattered *Betula lanata* and *Larix cajanderi* forests. In pure mats and with *Blepharostoma* sp., *Blepharostoma trichophyllum*,* Lophozia lantratoviae*,* Preissia quadrata*,* Trilophozia quinquedentata* and* Tritomaria scitula*. Kh-49-23-19, Kh-50-33-19, Kh-58-1-19.

***Mesoptychia rutheana*** (Limpr.) L. Söderstr. & Váňa – **26** – 91 m – Open wet hummocks and hollows in eutrophic hypnaceous swamp with sluggishly-flowing waters, developed over limestone. In pure mats and with *Aneura pinguis*. Kh-59-4-19, Kh-59-5-19, Kh-59-6-19.

***Metacalypogeia cordifolia*** (Stephani) Inoue – **17**, **18**, **22** – 82–154 m – Open to partly shaded moist cliffs in large riverside variously-faced cliff massifs with scattered *Betula lanata*. With *Bazzania denudata*,* Bazzania tricrenata*,* Blepharostoma* sp.,* Blepharostoma trichophyllum*,* Cephaloziella *sp.,* Diplophyllum taxifolium*,* Douinia plicata*,* Herbertus *aff.* buchii*,* Lepidozia reptans*,* Marsupella emarginata*,* Plagiochila porelloides*,* Solenostoma rossicum*,* Sphenolobus minutus* and* Tritomaria exsecta*. Kh-49-4-19, Kh-49-7-19, Kh-49-20-19, Kh-50-19-19, Kh-50-36-19, Kh-50-42-19, Kh-54-1-19.

***Metzgeria pubescens*** (Schrank) Raddi –**18**, **19** – 129–780 m – Open moist to mesic cliffs and their crevices at ridgeline surrounded by subalpine vegetation and cliff-like massif at riverside in low elevation, once on thin *Sorbus* trunk base in scattered *Betula lanata* forest. In pure mats or with *Barbilophozia barbata*,* Diplophyllum taxifolium*,* Douinia plicata* and* Plagiochila porelloides*. Kh-50-13-19, Kh-50-38-19, Kh-51-11-19, Kh-56-17-19.

***Mylia anomala*** (Hook.) Gray – **12** – 1109 m – Over humus on wet mossy stream bank in moist mossy tundra in alpine belt. With *Fuscocephaloziopsis pleniceps* and* Protochilopsis grandiretis*. Kh-44-3-19, Kh-44-10-19.

***Mylia verrucosa*** Lindb. – **15**, **23** – 118–143 m – Partly shaded moist decaying wood and, rarely, humus on the trail-side in *Picea ajanensis *pure or with *Betula lanata* admixture forests. With *Anastrophyllum michauxii*,* Blepharostoma *sp.,* Calypogeia suecica*,* Cephalozia bicuspidata*,* Crossocalyx hellerianus*,* Fuscocephaloziopsis lunulifolia*,* Lepidozia reptans*,* Lophozia guttulata*,* Lophozia *sp.,* Lophozia ventricosa *and * Schistochilopsis incisa*. Kh-47-3-19, Kh-47-17-19, Kh-55-1-19, Kh-55-11-19.

***Nardia geoscyphus*** (De Not.) Lindb. – **15** – 143 m – Open moist cliffs near stream and humus covering rocks on slope in *Picea ajanensis* dominating forest in narrow valley. With *Anthelia juratzkana*,* Cephalozia bicuspidata*,* Diplophyllum taxifolium*,* Marsupella boeckii*,* Pleurocladula albescens Pseudolophozia sudetica*. Kh-47-7-19, Kh-47-20-19, Kh-47-21-19.

***Neoorthocaulis binsteadii*** (Kaal.) L. Söderstr., De Roo & Hedd. – **6**, **11** – 904–1148 m – Open moist *Sphagnum* mats in alpine and subalpine belts, in the latter case surrounded by mosaic of *Duschekia* and *Pinus pumila* clumps and mountain tundra vegetation on steep N-facing slope. With *Calypogeia neogaea*,* Cephalozia *sp.,* Lophozia *sp. and* Sphenolobus minutus*. Kh-38-5-19, Kh-43-8-19.

***Pellia endiviifolia*** (Dicks.) Dumort. – **25** – 22 m – Open moist humus along stream with many limestone boulders in the streambed in open *Larix* forest in the valley. In pure mats. Kh-58-2-19.

***Pellia neesiana*** (Gottsche) Limpr. – **15** – 143 m – Partly shaded moist humus on slope in *Picea ajanensis* dominating forest in narrow valley. With *Blepharostoma trichophyllum* ,* Plagiochila porelloides* and* Scapania* sp. Kh-46-8-19, Kh-47-6-19.

***Peltolepis quadrata*** (Saut.) Müll. Frib. – **18** – 129–154 m – Open wet cliff crevice in large N-facing conglomerate cliffs with scattered *Betula lanata* along riverside. With *Plagiochila porelloides*. Kh-56-4-19.

***Plagiochila arctica*** Bryhn & Kaal. – **9** – 1484 m – Moist humus moistened by basic reaction percolating water on steep N-facing slope in alpine belt. With *Scapania crassiretis*. Kh-41-8-19.

***Plagiochila porelloides*** (Torr. ex Nees) Lindenb. – **1**, **14**, **16**, **18**, **23**, **27** – 5–154 m – Open to partly shaded moist to wet boulders (also those along watercourses) and humus on slopes, mesic cliffs, rarely decaying wood, in various types of forests (dominated by *Larix cajanderi* and *Picea ajanensis*, with a certain admixture of *Betula lanata*), subalpine clumps of *Duschekia* and *Pinus pumila*, also tundra-like communities along seacoast, once on moist hummock on the windy meadow in a small saddle. In pure mats or with *Bazzania tricrenata*,* Blepharostoma* sp., *Blepharostoma trichophyllum*, *Conocephalum salebrosum*,* Diplophyllum taxifolium*,* Frullania subarctica*,* Herbertus *aff.* buchii*,* Metacalypogeia cordifolia*,* Metzgeria pubescens*,* Pellia neesiana*,* Peltolepis quadrata*,* Scapania* sp.,* Solenostoma rossicum* and*Tritomaria exsecta*. Kh-33-1-19, Kh-46-9-19, Kh-48-3-19, Kh-50-50-19, Kh-55-4-19, Kh-56-2-19, Kh-58-5-19, Kh-58-7-19, Kh-60-2-19.

***Plagiochila satoi*** S. Hatt. – **17**, **24** – 24–82 m – Open moist humus between limestone outcrops on steep slope surrounded by tundra-like community developed as the result of severe wind conditions. With *Mesoptychia gillmanii* and* Preissia quadrata*. Kh-49-8-19, Kh-57-20-19.

***Pleurocladula albescens*** (Hook.) Grolle – **12**, **15** – 143–1109 m – Open humus banks and cliffs near stream; from low to high elevations: in *Picea ajanensis* forest in narrow valley and moist mossy tundra. With *Anthelia juratzkana*,* Blepharostoma* sp., *Diplophyllum taxifolium*,* Gymnocolea inflata*,* Marsupella boeckii*,* Marsupella emarginata*,* Nardia geoscyphus*,* Protochilopsis grandiretis*,* Pseudolophozia debiliformis*,* Pseudolophozia sudetica*,* Scapania parvifolia*,* Scapania* sp. and *Trilophozia quinquedentata*. Kh-44-7-19, Kh-44-9-19, Kh-47-20a-19.

***Porella vernicosa*** Lindb. – **18** – 129–154 m – Open mesic cliff crevice in large riverside N-facing cliffs with scattered *Betula lanata* in low elevations. In pure mats. Kh-50-2-19, Kh-50-3-19.

***Prasanthus suecicus*** (Gottsche) Lindb. – **9** – 1484 m – Open wet humificated soil moistened by percolating waters of neutral reaction near small temporary stream in alpine belt with scattered vegetation, on steep N-facing slope. With *Anthelia juratzkana* and *Cephalozia bicuspidata*. Kh-41-6-19.

***Preissia quadrata*** (Scop.) Nees – **10**, **16**, **18**, **22**, **24**, **28** – 5–1478 m – Open to partly shaded moist cliff crevices (including those of limestone) filled with humus or fine soil, rarely open wet humus, moistened with percolating waters of neutral reaction, near temporary stream; in all elevation belts. In pure mats or with *Blepharostoma* sp., *Eocalypogeia schusteriana*,* Jungermannia afoninae*,* Mannia pilosa*,* Mesoptychia gillmanii*,* Mesoptychia heterocolpos*,* Plagiochila satoi*,* Scapania gymnostomophila*,* Trilophozia quinquedentata* and* Tritomaria quinquedentata*. Kh-42-2-19, Kh-48-2-19, Kh-50-14-19, Kh-50-18-19, Kh-50-30-19, Kh-54-2-19, Kh-57-21-19, Kh-61-3-19.

***Protochilopsis grandiretis*** (Lindb. ex Kaal.) A.V. Troitsky, Bakalin & Fedosov – **6**, **9**, **12** – 904–1484 m – Open moist peat on slope in subalpine belt amongst mosaic of *Duschekia* and *Pinus pumila* clumps and mountain tundra vegetation on steep N-facing slope to stream. With *Cephaloziella varians*,* Cryptocolea imbricata*,* Fuscocephaloziopsis pleniceps*,* Gymnocolea inflata*,* Mylia anomala*,* Pleurocladula albescens*,* Riccardia palmata* and* Scapania *sp. Kh-38-1-19, Kh-41-9-19, Kh-44-3-19.

***Pseudolophozia debiliformis*** (R.M. Schust. & Damsh.) Konstant. & Vilnet. – **12** – 1109 m – Over humus on wet mossy stream bank in moist mossy tundra. With *Anthelia juratzkana* and* Pleurocladula albescens*. Kh-44-1-19.

***Pseudolophozia sudetica*** (Nees ex Huebener) Konstant. & Vilnet – **4**, **7**, **12**, **15**, **17**, **21** – 82–1261 m – Open to partly shaded moist cliffs and boulders, including those near stream and waterfall canyon; in open *Larix* *cajanderi* forest or scattered *Betula lanata* stands. With *Anthelia juratzkana*,* Blepharostoma *sp.,* Cladopodiella francisci*,* Diplophyllum taxifolium*,* Gymnomitrion concinnatum*,* Gymnomitrion corallioides*,* Marsupella boeckii*,* Marsupella emarginata*,* Nardia geoscyphus*,* Pleurocladula albescens*,* Scapania crassiretis*,* Scapania *sp.,* Schistochilopsis incisa* and* Trilophozia quinquedentata*. Kh-36-7-19, Kh-39-3-19, Kh-44-9-19, Kh-47-21-19, Kh-49-19a-19, Kh-53-4-19.

***Pseudotritomaria heterophylla*** (R.M. Schust.) Konstant. & Vilnet – **9**, **10** – 1478–1484 m – Open moist sandy soil and humus moistened by percolating water of basic reaction, also those near small temporary stream; in alpine belt with scattered vegetation. With *Anthelia juratzkana*,* Cryptocolea imbricata* and* Scapania parvifolia*. Kh-41-5-19, Kh-42-3a-19.

***Ptilidium ciliare*** (L.) Hampe – **3**, **6**, **9**, **10**, **19** – 11–1484 m – Open mesic boulders and cliffs in forest and subalpine belts, rarely wet hollows between mossy hummock on slope amongst subalpine vegetation by a mosaic of *Duschekia* and *Pinus pumila* clumps and mountain tundra. In pure mats or with *Radula prolifera* and* Scapania crassiretis*. Kh-35-5-19, Kh-38-8-19, Kh-41-7-19, Kh-42-4-19, Kh-51-7-19.

***Ptilidium pulcherrimum*** (Weber) Vain. – **1**, **3**, **6**, **14**, **23** – 11–904 m – Partly shaded moist decaying wood in *Picea ajanensis* (sometimes with *Betula lanata* admixture) forests, *Pinus pumila* living branches in subalpine belt, rarely moist humus near stream in *Larix cajanderi* forest with *Pinus pumila* understorey. In pure mats or with *Crossocalyx hellerianus*. Kh-33-2-19, Kh-35-1-19, Kh-46-5-19, Kh-55-9-19.

***Radula complanata*** (L.) Dumort. – **18**, **23** – 129–154 m – Partly shaded moist decaying wood in *Picea ajanensis* with scattered *Betula lanata* forest, open to partly shaded moist cliffs and their crevices in large N-facing conglomerate cliffs with scattered *Betula lanata* along riverside. In pure mats. Kh-50-15-19, Kh-55-6-19, Kh-56-23-19.

***Radula prolifera*** Arnell – **10** – 1478 m – Open sandy soil moistened by percolate waters of basic reaction on slope with scattered vegetation in alpine belt. With *Anthelia juratzkana*,* Cryptocolea imbricata* and* Ptilidium ciliare*. Kh-42-4-19.

***Reboulia hemisphaerica*** (L.) Raddi subsp. ***orientalis*** R.M. Schust. – **18**, **21** – 129–235 m – Open to partly shaded moist cliff crevices and caves in large rocky outcrops along riverside and waterfall canyon at low elevation, surrounded by scattered *Betula lanata* stands. The second of the cited specimens is sterile and referred to this subspecies tentatively. In pure mats. Kh-50-9-19, Kh-53-3-19.

***Riccardia palmata*** (Hedw.) Carruth. – **9** – 1484 m – Over humus moistened by percolating waters of neutral reaction, near temporary stream on N-facing slope in alpine belt with scattered vegetation. With *Cephaloziella varians*,* Cryptocolea imbricata*,* Fuscocephaloziopsis pleniceps*,* Scapania* sp. and *Protochilopsis grandiretis*. Kh-41-9-19.

***Riccia bifurca*** Hoffm. – **17**, **18** – 82–154 m – Open mesic cliff crevice in large riverside N-facing cliffs surrounded by scattered *Betula lanata*. With *Mannia* cf. *sibirica*. Kh-48-1-19, Kh-49-13-19, Kh-50-3-19. Kh-51-3-19.

***Riccia sorocarpa*** Bisch. – **17** – 82 m – Open moist humus filling cliff crevice in large riverside S-facing cliffs surrounded by scattered *Betula lanata*. With *Cephaloziella divaricata* and* Mannia sibirica*. Kh-49-3-19.

***Sauteria alpina*** (Nees) Nees – **18** – 129–154 m – Open moist cliff crevices also those filled with humus in large N-facing conglomerate riverside cliffs surrounded by scattered *Betula lanata*. In pure mats and with *Eremonotus myriocarpus* and *Solenostoma obscurum*. Kh-50-12-19, Kh-56-9-19, Kh-56-10-19, Kh-56-11-19, Kh-56-12a-19.

***Scapania crassiretis*** Bryhn – **7**, **9**, **18**, **22** – 119–1484 m – Open to partly shaded moist to wet cliffs and their crevices, rarely moist humus on slope, only along watercourses (although sometimes at a distance from the running water); in alpine belt with scattered vegetation, rarely in open places on riverside rocky outcrops in scattered forest. In pure mats or with *Diplophyllum taxifolium*,* Frullania subarctica*,* Gymnomitrion corallioides*,* Herbertus *aff.* buchii*,* Plagiochila arctica*,* Pseudolophozia sudetica*,* Ptilidium ciliare* and* Trilophozia quinquedentata*. Kh-39-1-19, Kh-39-7-19, Kh-39-13-19, Kh-41-7-19, Kh-50-1-19, Kh-50-45-19, Kh-50-54-19, Kh-54-3-19.

***Scapania cuspiduligera*** (Nees) Müll. Frib. – **25** – 22 m – Partly shaded moist humus along stream, with many limestone boulders in the stream bed in open *Larix* forest. In pure mats. Kh-58-6-19.

***Scapania gymnostomophila*** Kaal. – **18**, **24** – 34–154 m – Humus covering rocks and cliff crevices, only those in limestone and basic conglomerates; at low elevation, surrounded by tundra-like community developed due to severe wind conditions near seacoast or by scattered *Betula lanata* over large riverside N-facing cliffs. With *Blepharostoma sp.*,* Eocalypogeia schusteriana*,* Jungermannia afoninae*,* Lophozia lantratoviae*,* Mannia pilosa*,* Mesoptychia badensis*,* Preissia quadrata* and* Trilophozia quinquedentata*. Kh-50-1-19, Kh-50-51-19, Kh-56-15-19, Kh-56-22-19, Kh-57-7-19, Kh-57-10-19, Kh-57-18-19, Kh-57-22-19.

***Scapania hyperborea*** Jørg. – **6** – 904 m – Open wet cliff on N-facing slope to stream in subalpine belt amongst mosaic of *Duschekia* and *Pinus pumila* clumps and mountain tundra vegetation. In pure mats. Kh-38-10a-19.

***Scapania irrigua*** (Nees) Nees – **13**, **14**, **15**, **21** – 54–1139 m – Open to partly shaded stones and humus covering rocks along streams and in waterfall canyons; from low elevations with *Picea ajanensis* dominating forests (also those with admixture of *Betula lanata*) to moist mountain moss-dwarf shrub tundra. In pure mats or with *Cephalozia bicuspidata*,* Jungermannia pumila* and* Nardia* cf. *geoscyphus*. Kh-45-1-19, Kh-46-2-19, Kh-47-11-19, Kh-53-8-19.

***Scapania microdonta*** (Mitt.) Müll.Frib – **5**, **8**, **19** – 418–1328 m – Open to partly shaded mesic crevices in cliffs and between boulders in gravelly barrens in alpine and subalpine belts. In pure mats or with *Barbilophozia barbata*,* Sphenolobus minutus*,* Sphenolobus saxicola* and* Tetralophozia setiformis*. Kh-37-3-19, Kh-40-8-19, Kh-51-1-19.

***Scapania mucronata*** H. Buch – **1**, **3**, **4**, **17**, **18** – 11–377 m – Open moist cliffs and boulders covered with thin layer of humificated soil, rarely humus on trail sides; in low elevations collected in scattered *Betula lanata* forest surrounding riverside cliffs and *Larix* *cajanderi* forest in narrow valley; in subalpine belt collected in scattered *Duschekia* and *Pinus pumila* clumps on steep slope. With *Blepharostoma *sp.,* Diplophyllum sibiricum*,* Diplophyllum taxifolium*,* Marsupella emarginata*,* Schistochilopsis sp.*,* Solenostoma obscurum* and* Tritomaria exsecta*. Kh-33-4-19, Kh-35-6-19, Kh-36-6-19, Kh-49-19-19, Kh-56-6-19.

***Scapania paludicola*** Loeske & Müll. Frib. – **6** – 904 m – Humus on the bank of sluggishly-flowing stream in moist moss-dwarf shrub tundra. In pure mats. Kh-45-1a-19.

***Scapania parvifolia*** Warnst. – **1**, **9**, **11**, **12** – 11–1484 m – Open (or, rarely, partly shaded) moist sandy, clayish and humificated soil on steep slopes (also those going to streams); mostly in alpine belt in scattered dwarf shrub-moss tundras, also those developed in percolating waters, once in subalpine belt amongst *Duschekia* and *Pinus pumila* clumps on the slope ca. 30° steepness. With *Calypogeia integristipula*,* Calypogeia muelleriana*,* Cephalozia bicuspidata*,* Lophozia *sp.,* Pleurocladula albescens*,* Pseudotritomaria heterophylla*,* Sphenolobus minutus* and* Trilophozia quinquedentata*. Kh-33-9-19, Kh-41-2-19, Kh-43-5-19, Kh-44-7-19.

***Scapania rufidula*** Warnst. – **3**, **18**, **20** – 129–294 m – Open wet boulders near streams, rarely sandy stream banks; in floodplain (dominating by *Populus suavealons* and *Salix udensis*), low elevation *Picea ajanensis* and *Betula lanata* and their combinations dominating forests. In pure mats or with *Blepharostoma *sp. and* Lophozia lantratoviae*. Kh-35-4-19, Kh-35-13-19, Kh-50-11-19, Kh-52-1-19, Kh-56-7-19, Kh-56-8-19.

***Scapania sphaerifera*** H. Buch & Tuom. – **19** – 780 m – Open mesic cliff crevice at the mountain ridge just above crooked forest line. With *Barbilophozia hatcheri*,* Sphenolobus saxicola* and* Tetralophozia setiformis*. Kh-51-9-19.

***Schistochilopsis incisa*** (Schrad.) Konstant. s.l. – **1**, **4**, **6**, **7**, **14**, **15**, **17**, **18**, **23** – 11–1261 m – Party shaded moist decaying wood and humus covering cliffs in *Picea ajanensis* with various proportions of admixture of *Betula lanata* forests, as well as scattered *Betula lanata* stands surrounding large riverside cliffs; moist to wet cliffs and their crevices and moist boulders near streams in subalpine (with mosaic of *Duschekia* and *Pinus pumila* clumps and mountain tundra vegetation on steep N-facing slope) and alpine (scattered tundra vegetation amongst gravelly barrens) belts, once found in floodplain forest dominating by *Populus suavealons* and *Salix udensis*. With *Blepharostoma *sp.,*Calypogeia integristipula*,* Cephalozia bicuspidata*,* Diplophyllum taxifolium*,* Douinia plicata*,* Fuscocephaloziopsis lunulifolia*,*Lepidozia reptans*,*Marsupella boeckii*,* Marsupella emarginata*,* Mylia verrucosa*,* Pseudolophozia sudetica*,* Solenostoma obscurum*,* Sphenolobus minutus*,* Trilophozia quinquedentata* and *Tritomaria exsecta*. Kh-38-13-19, Kh-39-8-19, Kh-46-13-19, Kh-47-2-19, Kh-47-3-19, Kh-49-15-19, Kh-55-1-19, Kh-56-3-19.

***Schljakovia kunzeana*** (Huebener) Konstant. & Vilnet – **12** – 1109 m – Wet mossy bank of the stream in moist mossy tundra. With *Gymnocolea inflata*. Kh-44-12-19.

***Solenostoma*** cf.*** confertissimum*** (Nees) Schljakov. – **15** – 143 m – Partly shaded moist boulder on slopes in mostly *Picea ajanensis* forest in narrow valley. With *Mesoptychia *aff. *heterocolpos*. Kh-47-10-19.

***Solenostoma obscurum*** (A. Evans) R.M. Schust. – **4**, **15**, **18**, **20**, **21** – 129–377 m – Open to partly shaded moist to wet boulders and cliffs along streams and river, also waterfall canyons in *Populus suaveolens* and *Salix udensis* floodplain forests and *Picea ajanensis* dominating forests on slopes. In pure mats and with *Anthelia juratzkana*,* Cephalozia bicuspidata*,* Diplophyllum taxifolium*,* Eremonotus myriocarpus*,* Sauteria alpina*,* Scapania mucronata*,* Scapania *sp.,* Schistochilopsis incisa*,* Schistochilopsis incisa* and* Trilophozia quinquedentata*. Kh-36-5-19, Kh-47-22-19, Kh-47-27-19, Kh-50-53-19, Kh-52-2a-19, Kh-53-5-19, Kh-56-6-19.

***Solenostoma rossicum*** Bakalin & Vilnet – **18** – 129–154 m – Open moist cliffs and their crevices partly filled with humus in large N-facing riverside conglomerate cliffs with scattered *Betula lanata*. With *Bazzania tricrenata*,* Cephaloziella *sp.,* Herbertus *aff.* buchii*,* Metacalypogeia cordifolia*,* Plagiochila porelloides*,* Sphenolobus minutus* and* Tritomaria exsecta*. Kh-50-20-19, Kh-56-20-19.

***Sphenolobus minutus*** (Schreb.) Berggr. – **1**, **3**, **5**, **6**, **8**, **9**, **11**, **17**, **18** – 11–1484 m – Partly shaded mesic boulders, open moist cliffs, dense *Sphagnum* cushions, rarely partly shaded moist clayish soil on steep slopes; from low elevation floodplain and *Picea ajanensis* forests via krumholtz to alpine belt with scattered vegetation represented by dwarf shrub-lichen tundra and *Sphagnum lenense* mats on percolate water openings, surrounded by gravelly barrens. In pure mats or with *Anthelia juratzkana*,* Bazzania tricrenata*,* Blepharostoma *sp.,* Calycularia laxa*,* Calypogeia integristipula*,* Calypogeia muelleriana*,* Calypogeia *sp.,* Cephalozia bicuspidata*,* Cephaloziella *sp.,* Diplophyllum taxifolium*,* Douinia plicata*,* Gymnomitrion corallioides*,* Herbertus *aff.* buchii*,* Lepidozia reptans*,* Lophozia silvicoloides*,* Lophozia *sp.,* Marsupella emarginata*,* Metacalypogeia cordifolia*,* Neoorthocaulis binsteadii*,* Scapania microdonta*,* Scapania parvifolia*,* Schistochilopsis incisa*,* Solenostoma rossicum* and* Tetralophozia setiformis*. Kh-33-6-19, Kh-35-12-19, Kh-35-15-19, Kh-37-3-19, Kh-38-6-19, Kh-39-12-19, Kh-40-6-19, Kh-41-2-19, Kh-43-3-19, Kh-43-7-19, Kh-49-4-19, Kh-50-37-19, Kh-56-21-19.

***Sphenolobus saxicola*** (Schrad.) Steph. – **5**, **7**, **8**, **11**, **19** – 418–1328 m – Open mesic cliff crevices, also those filled with soil, rarely dense *Sphagnum* cushions; in alpine belt with scattered vegetation amongst gravelly barrens or ridgeline cliffs. In pure mats or with *Barbilophozia hatcheri*,* Calycularia laxa*,* Lophozia *sp.,* Scapania microdonta*,* Scapania sphaerifera* and* Tetralophozia setiformis*. Kh-37-2-19, Kh-39-4-19, Kh-40-8-19, Kh-43-12-19, Kh-51-5-19.

***Tetralophozia setiformis*** (Ehrh.) Schljakov – **5**, **6**, **8**, **11**, **19** – 418–1328 m – Mesic cliffs on ridgeline, crevices between boulders in gravelly barrens, rarely moist dense *Sphagnum lenense* cushions; only in alpine belt. In pure mats or with *Anthelia juratzkana*,* Barbilophozia hatcheri*,* Gymnocolea inflata*,* Scapania microdonta*,* Scapania sphaerifera*,* Sphenolobus minutus* and* Sphenolobus saxicola*. Kh-37-1-19, Kh-38-10-19, Kh-40-6-19, Kh-43-10-19, Kh-51-6-19.

***Trilophozia quinquedentata*** (Huds.) Bakalin – **2**, **4**, **12**, **15**, **18**, **19**, **22** – 119–1109 m – Open to partly shaded moist to wet cliffs and their crevices, boulders, also those near streams, open moist humus on stream banks; from low elevation in various forests, through subalpine belt in scattered shrubby clumps to alpine belt in scattered vegetation surrounded by gravelly barrens and ridgeline cliffy massifs. In pure mats or with *Bazzania tricrenata*,* Blepharostoma sp.*,* Calycularia laxa*,* Cephalozia bicuspidata*,* Diplophyllum taxifolium*,* Frullania davurica*,* Lophozia lantratoviae*,* Marsupella emarginata*,* Mesoptychia heterocolpos*,* Pleurocladula albescens*,* Preissia quadrata*,* Pseudolophozia sudetica*,* Scapania crassiretis*,* Scapania gymnostomophila*,* Scapania parvifolia*,* Schistochilopsis incisa*,* Solenostoma obscurum* and* Tritomaria scitula*. Kh-34-4-19, Kh-36-1-19, Kh-44-6-19, Kh-47-22-19, Kh-46-11-19, Kh-50-22-19, Kh-51-8-19, Kh-54-3-19, Kh-56-14-19.

***Tritomaria exsecta*** (Schmidel ex Schrad.) Schiffn. ex Loeske – **4**, **18**, **23** – 118–377 m – Open moist cliff crevices partly filled with humus, partly shaded humus covered boulders near streams; low elevation riverside cliff-like massifs with scattered *Betula lanata* and floodplain forests dominated by *Populus suavealons* and *Salix udensis*. With *Bazzania tricrenata*,* Blepharostoma *sp.,* Lepidozia reptans*,* Lophozia *sp.,* Metacalypogeia cordifolia*,* Plagiochila porelloides*,* Scapania mucronata*,* Schistochilopsis incisa* and* Solenostoma rossicum*. Kh-36-6-19, Kh-50-21-19, Kh-55-1-19.

***Tritomaria scitula*** (Taylor) Jørg. – **18** – 129–154 m – Open moist cliff crevice in large riverside N-facing cliffs with scattered *Betula lanata*. With *Blepharostoma *sp.,* Lophozia lantratoviae*,* Mesoptychia heterocolpos* and* Trilophozia quinquedentata*. Kh-50-32-19.

## Discussion

### Taxonomic diversity and ecological traits

The total number of revealed taxa (118 species, including one criptic taxon and 1 subspecies) is quite high for relatively brief studies of the local hemiarctic flora. This is likely a consequence of co-action of habitat diversity (of geological, orographic and climatic origin) and historical reasons (the position of explored area at the migration route between Northeast Asia and East Asia). Meanwhile, not all revealed taxa are listed in the checklist. There are several specimens which were not identified convincingly. These doubtfully identified specimens require a molecular-genetic study to find the name (some of them may belong to undescribed taxa). These unlisted taxa may potentially provide certain additional peculiarities of the studied flora.

Relatively few in number are ‘forest’ taxa, represented here by boreal and hemiboreal species (distributed mostly in corresponding zones of the Northern Hemisphere). Several of them were found on decaying wood in *Picea ajanensis* forests (less in number are epixylous taxa in *Larix cajanderi* forests): *Fuscocephaloziopsis leucantha* and *Mylia verrucosa* are locally abundant, *Crossocalyx* *hellerianus* is rare, *Anastrophyllum michauxii*, *Calypogeia suecica* and *Radula complanata* (also recorded from the cliff) were found only once. *Bazzania denudata* is rare and found on rocky substrates, only in the localities near the seacoast. *Frullania bolanderi*, apparently, is quite frequent in floodplain communities, on the trunks of *Salix udensis* and *Betula lanata*. Rarely observed are *Lophocolea heterophylla* (floodplain forests only) and *Lophozia ventricosa* (also found in *Picea–Betula* forest).

Two taxa complete the epiphytes in the area: *Frullania bolanderi* and *Ptilidium pulcherrimum*. The latter is widespread on tree trunks and thick branches in tall and crooked forest communities. Some taxa, commonly not limited to the forest communities in other hemiarctic floras, are exclusively forest taxa in the explored area: *Fuscocephaloziopsis lunulifolia*, *Lepidozia reptans*, *Lophocolea minor*, *Lophozia lantratoviae*, *L. silvicola*, *Scapania irrigua*, *S. mucronata*, *S. rufidula *and *Solenostoma obscurum*. *Douinia plicata* is found in all belts and is abundant in true forests and krummholtz. *Larix cajanderi* forests are poor in taxa (excluding liverwort flora of watercourses and other habitats only slightly related to the larch forest itself). Their dryness predict occurrence of meso-xerophytes, like *Barbilophozia barbata*. The species previously identified as *Blepharostoma brevirete* (Bryhn & Kaal.) Vilnet & Bakalin (*Blepharostoma* sp. (Kh-58-4-19) in the annotated checklist) was found in open *Larix* forest and was re-identified by genetic methods as a different, not yet described (tentatively cryptic) hybrid of *B. trichophyllum* and an unknown taxon (Bakalin et al. 2020a). It is morphologically similar to *B. brevirete*.

The krummholtz (*Pinus pumila* and *Duschekia fruticosa* thickets) houses a few liverwort taxa and have no specific flora. Besides *Ptilidium pulcherrimum* that is the commonest species in these communities, a whole series of species are found here, descending into crooked forests from the alpine belt along watercourses, i.e. by intrazonal habitats. On the contrary, examples of taxa that would join forests and krumholtz, but would be absent in alpine belt are few: *Lophozia silvicoloides*, *Ptilidium ciliare* and *Scapania mucronata – *their absence from the alpine belt may be the simple result of undercollecting, since all those listed are common inhabitants of alpine belts in other parts of the eastern Paleoarctic.

The arctic-montane acidophilous (or neutrotolerant) species were quite numerous. Widely distributed, though not confined to, but more frequent in the alpine belt, were *Anthelia juratzkana*, *Gymnomitrion corallioides*, *Pleurocladula albescens*, *Pseudolophozia sudetica*, *Scapania* *microdonta*, *Sphenolobus saxicola* and *Tetralophozia setiformis*. Other taxa were sparse and occurred locally in upper elevations: *Cephaloziella varians* and *Gymnomitrion concinnatum*. Several species were restricted to the high altitudes in the upper reaches of the axial part of Dzhugdzhur Range: *Gymnomitrion commutatum*, *Prasanthus* *suecicus* and *Protochilopsis grandiretis* were all very rare in the explored area. *Isopaches bicrenatus* (that is not, strictly speaking, purely arctic-montane species) was found only on solifluction spots in the axial part of Dzhugdzhur Range. *Neoorthocaulis binsteadii* was very common on *Sphagnum* cushions in the axial part. *Riccardia palmata* that was rather expected to be found on decaying wood in lower altitudes, was found only in the alpine belt in tundra environments. Similarly, mainly swampy *Scapania paludicola* and *Schljakovia kunzeana* were found in moist moss-dwarf shrub tundra near streams. Rare were *Diplophyllum sibiricum*, *Jungermannia afoninae*, *Marsupella boeckii* and *M. emarginata*. *Scapania sphaerifera*, expected in the axial part, was found at the highest elevations in Pribrezhny Range in the coastal part of the area only.

A limited number of species were common in all altitudinal belts: *Calypogeia integristipula*, *Lophozia guttulata* (the specimens referred to this species were gathered in mountain tundra and may represent another taxon not yet described), *L. lantratoviae*, *Scapania crassiretis* and *Sphenolobus minutus*. Sparsely, although occurring across all belts, were *Metzgeria pubescens*, *Plagiochila porelloides*, *Preissia quadrata*, *Scapania parvifolia*, *S. rufidula *(restricted to the axial part of Dzhugdzhur Range), *Schistochilopsis incisa*, *Sphenolobus minutus*, *Trilophozia quinquedentata* and *Tritomaria exsectiformis*.

The abundance of Cretaceous sediments of basic reactions (limestone and its derivates) has determined the wide distribution of basiphilous taxa accounting for about 10% of the liverwort flora. The presence of basiphilous taxa is a peculiar feature of studied flora in comparison with the vast majority of other local floras in Hemiarctic Pacific Asia. Striking examples of basiphilous taxa include *Eocalypogeia schusteriana*, four species of *Mannia*, three species of *Mesoptychia* (*M. badensis*, *M. gillmanii*, *M. rutheana*), *Peltolepis quadrata*, *Pseudotritomaria heterophylla*, *Sauteria alpina*, *Scapania* *cuspiduligera* and *S. gymnostomophila*. *Conocephalum salebrosum* should be conditionally referred to the group of basiphilous taxa in the investigated area since it was found only in areas of distribution of limestone and neutral metamorphic rocks. *Mesoptychia heterocolpos* is also not the obligate basiphilous taxon; however, it was found only in the area where basic rocks are distributed. Three species, *Cryptocolea imbricata*, *Plagiochila arctica* and *Radula prolifera* although, were found in the axial part of Dzhugdzhur Range that is predominantly acidic, but restricted there to the places with a pronounced neutrophilic flora (including from vascular plants, for example, *Salix berberifolia* Pall.) and developed near basic reaction percolating water openings.

The rocky outcrop liverwort flora in lower altitudes usually is not liverwort-rich, with one striking exception. In the course of our works, we found a large riverside cliff-like massif on the right bank of the Unych’ya River in its middle course (56°25'07.3"N 137°56'54.7"E) that is very peculiar in its geological and ecological respect (Fig. 8a). The cliff area has a length of about 100 m along the riverbed and has a height of up to 50 m from the riverbed to the top. These are conglomerate rocks that unite both basic and acidic metamorphic 'pebbles' (expected only due to the bryophyte inhabitants of the rocky particles as no special chemical analyses were conducted there) (Fig. 8b). Ecologically, these cliff walls also provide a variety of moisture conditions (from very wet to very dry) combined with variations in illumination and various niches and caves. Besides, these cliffs are covered by scattered *Betula lanata* trees – in this case, by enriching potential habitat diversity by shading as well. The liverwort diversity occurred there covers about 40% of the entire explored flora, although with the vast majority of taxa found also in other complexes. *Bazzania tricrenata*, *Frullania davurica* and *Herbertus dicranus* were very common there, while *Jungermannia afoninae* and *Metacalypogeia cordifolia* were sparse. Several taxa, including *Erimonotus myriocarpus*, *Frullania ignatovii*,* F. subarctica*, *Lejeunea alaskana*,* Peltolepis quadrata*, *Porella vernicosa*,* Reboulia hemisphaerica*,* Sauteria alpina*, *Solenostoma rossicum* and *Tritomaria scitula* were found only in the mentioned cliff-like massif. Moreover, for some of the listed species (*Frullania ignatovii*,* F. subarctica* and *Lejeunea alaskana*), this location is very unusual, taking into account the low elevation that formally belongs to the forest belt. The striking trait of the liverwort complex in the described massif is the occurrence within the same locality of the species belonging to different flora elements: it is especially pronounced in the ‘pair’ *Metacalypogeia cordifolia* and *Eocalypogeia schusteriana* occurring in adjacent patches of the same cliff. The chemical composition diversity results in an almost artificial mixture of joint occurrence of acidophilous and basiphilous taxa, like *Bazzania denudata* growing together with *Tritomaria scitula*. Taking into account the total diversity and peculiarity of taxonomic composition, this cliff riverside complex deserves protection as a natural monument of regional significance.

The formerly (and on-going) migration of liverworts by the mountains along the western coast of the Sea of Okhotsk in view of possible connections between East Asian and North-East Asian floras may be well illustrated by the occurrence of mainly Beringian *Cryptocolea imbricata*, *Eocalypogeia schusteriana*, *Frullania ignatovii*, *Frullania subarctica* and *Fuscocephaloziopsis pachycaulis* found in the area. All of them were recorded also south of the explored area. 'Southern', mainly temperate East Asian elements were represented by a lesser number of taxa, including *Metacalypogeia cordifolia*, *Porella vernicosa* and *Mylia verrucosa*. For the first two taxa, the studied locality is the northernmost outpost of worldwide distribution. *Calypogia neogaea* – mainly East Asian–North American taxon, occurring in the areas adjacent to the oceanic coast – had in Ayan surroundings the most ‘inland’ and the most northern occurrence in the Asian mainland.

Describing characteristic traits of the liverwort flora in the area treated, the absence of several taxa, forming ‘negative’ peculiarity, should be also taken into account. A large number of boreal ‘forest’ taxa were not found in the area: *Bazzania trilobata*, *Nowellia curvifolia*, *Neoorthocaulis* *attenuatus* and *Scapania apiculata*. For all listed, it was quite unexpected, because they are known not only southwards, but also north of Ayan surroundings. The same should be noted on the taxa commonly not limited by forest communities and absent from the explored area: *Chyloscyphus polyanthos*, *Geocalyx graveolens* and *Harpanthus flotovianus*. *Aneura pinguis* occurs very locally. *Calypogeia muelleriana* is very rare. Even so widespread in Holarctic, *Cephalozia bicuspidata* is quite rare. Very infrequent were *Diplophyllum taxifolium*, *Gymnocolea inflata* and *Mylia anomala*. Once found were *Marchantia polymorpha* and *Pellia neesiana*. Extremely rare (and observed in the axial part of the Dzhhugdzhur Range only) was *Nardia* *geoscyphus*. Certainly, the ‘absence’ of some taxa may be caused by the imperfections of the study, but for some taxa, another explanation may be necessary. It is difficult to expect that we have overlooked large-sized plants such as *Chiloscyphus* or so widespread in Northeast Asia, *Harpanthus flotovianus*. The rarity of *Cephalozia bicuspidata* and *Diplophyllum taxifolium,* as well as several other taxa is also surprising. The possible explanations may be in the wide distribution of basic and neutral rocks (blocking wide distribution of acidophilous taxa) and the wide distribution of neutrophilous and neutrotolerant taxa, with the latter also able to occupy the habitats potentially suitable for acidophilous taxa. A similar phenomenon was observed in the boreal zone in Sakhalin Island in the area with the evident dominance of limestone outcrops and neutral rocks (Bakalin et al. 2019).

The vast majority of taxa belong to the arctic-montane flora elements, although boreal taxa are also quite common in the flora. Therefore, the liverwort flora, in general, possesses a subarctic character (thus being the same as the vegetation). Using Whittaker (1960) terminology, the relatively high *gamma* diversity of the explored area is associated with low level of *alpha* diversity: each small site by itself possesses a few species, but the difference in the inhabitants’ composition of different sites (e.g. basic and acidic rocks) is quite strong. The combination of various flora elements and taxa of different ‘acidophilia’ results in taxonomically quite rich liverwort flora. Nevertheless, although our assumption on the taxonomic richness of the liverwort flora in the studied area was confirmed, the revealed diversity cannot be regarded as the final calculation. Moreover, taking into account the short duration of the studies, the data obtained by us should be considered as preliminary only and may be supplemented with many new findings in the further studies.

#### Phytogeographic implications

**Figure 9.**
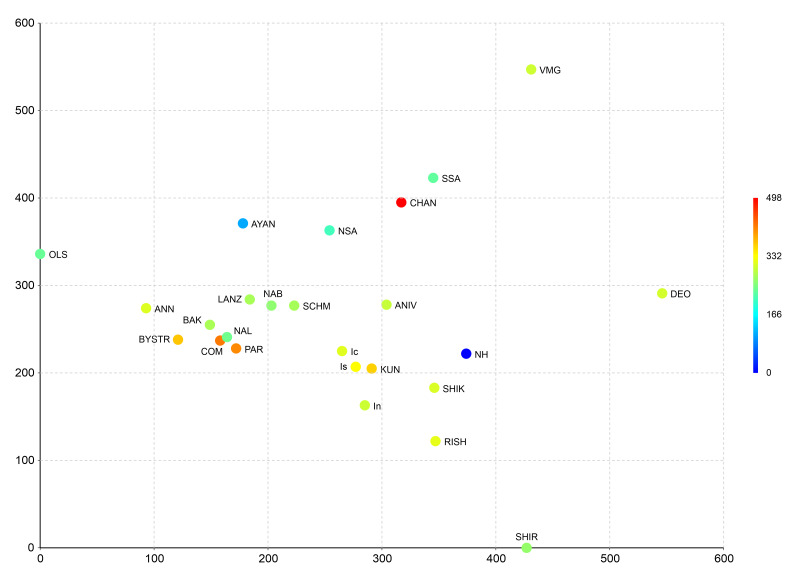
Compared flora distribution in DCA bubble chart (third axis is the colour gradient from deep blue to deep red).

**Figure 10.**
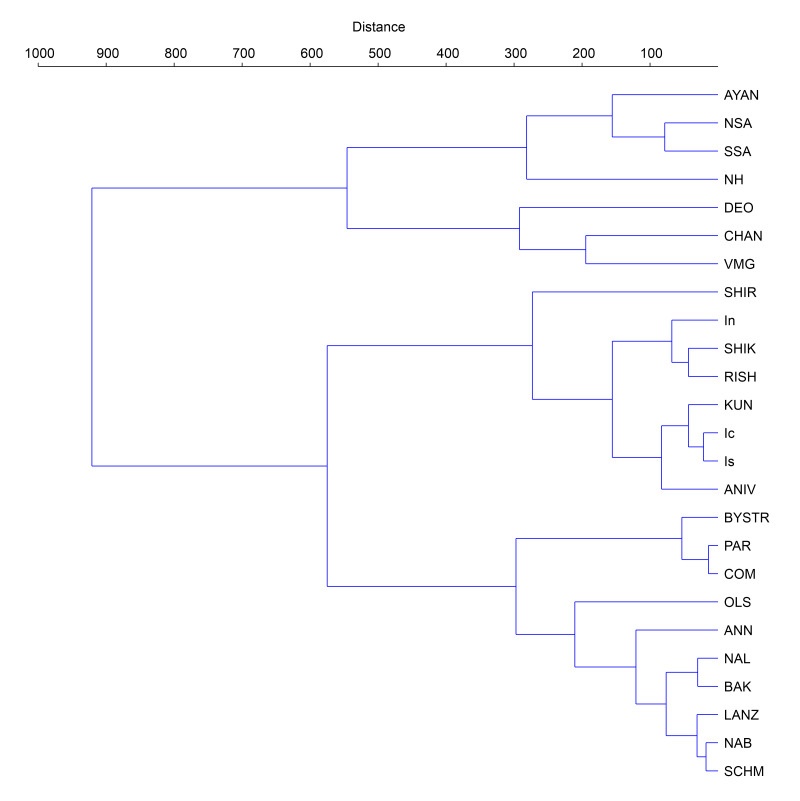
Hierarchical clustering (Ward’s method, Euclidean distance).

The DCA showed results in graphical form in Fig. 9, whereas Normalized numerical results are in Table 3. The hierarchical clustering in Fig. 10 (Ward’s method, Euclidean distance) has shown more or less similar results. Following the latter, the most related floras to Ayan surroundings are those in South and North Sikhote-Alin (coresponding to SSA and NSA). Geographically, they are not the nearest to Ayan (e.g. the floras in northern Sakhalin Island are two times nearer and are on more similar latitudes). This cluster is united then with NH (the northernmnost part of Hokkaido Island) and then with the cluster including East-Manchurian Mountains (VMG), Changbai Mts. (CHAN) and Deokgyu Mts. (DEO) in the central part of Korea.

This unexpected relationship to the southerly situated floras inspired us to check the analysis results by its comparison with the mean distances from Ayan flora revealed by DCA. Since the results of DCA analysis are points in three-dimensional space, it is convenient to use the Euclidean distance metric to compare the minimal distance between points. The normalized Euclidean distance between the Ayan flora and the nearest other flora is shown in Table 4 (the distance to the nearest flora is taken as a unit). The most closely-related flora is the flora of North Sikhote-Alin (NSA, over 800 km south from Ayan), then (as is quite understandable) the flora of Lanzhinskiye Mts., situated in North Okhotiya (430 km by direct line from Ayan). The next is the mountain flora of Nabilsky Range in the middle Sakhalin (NAB). The latter is followed by South Sikhote-Alin (SSA), Shiretoko in North Hokkaido (NH), Schmidt Peninsula in Northern Sakhalin (SCHM) (geographically the nearest, slightly over 400 km from Ayan) and Ol’skoye Basalt Plateau (OLS) in Kolyma Upland (over 1000 km distance).

As it could be seen from above placed description and also evident from Figs 9, 10, Ayan occupies the intermediate position between hemiboreal floras of continental mainland (Sikhote-Alin System) and hemiarctic continental and subcontinental floras in North Okhotiya and Kolyma Upland. The latter is a unique phenomenon of the flora equi-related to the conditionally ‘northern’ and ‘southern’ floras. Meantime, one more common feature may be revealed if looking precisely for the floras most related to Ayan: 1) Some of them: OLS, NSA and NAB provide the habitats for basiphilous plants (limestone or other basic substrates, like basalts). The Annachag Range (ANN), although nearer to Ayan than Ol'skoye Basalt Plateau (OLS), does not show these distinct relationships despite being entirely acidic (and the flora is composed by purely acidophilous plants). The relationships shown between Ayan and North Hokkaido (NH) may be an aberration we cannot explain definitely, although high elevations in the Island are the habitats for many arctic-alpine plants.

## Conclusion

Ayan flora is the phytogeographical link between East Asian hemiboreal and Northeast Asian hemiarctic floras and shows relationships to both. Ayan surroundings were liverwort ‘terra incognita’ before the present study. Therefore, the conducted research is our attempt to reduce ‘blank spots’ and ‘lost diversity’ in liverwort flora of East Asia (Klimova and Bakalin 2019). Moreover, two additional arguments in favour of such kind of research are noticeable: 1) the collected material may be the basis for taxonomic revisions which rely on an integrative approach and understanding of the plant genetic diversity in the land; 2) the study of floras occupying an intermediate position between two vegetation zones (like Ayan) confirms the gradual changes from subarctic to hemiboreal floras – the fact commonly estimated, but infrequently tested in bryological researches.

## Supplementary Material

106E54C2-7969-5000-8EA8-8D136A7B5173 10.3897/BDJ.9.e65199.suppl1 Supplementary material 1 The distribution of liverworts in compared floras Data type Occurrences File: oo_525377.xlsx https://binary.pensoft.net/file/525377 Bakalin V, Klimova K, Bakalin D, Choi SS

## Figures and Tables

**Figure 2a.**
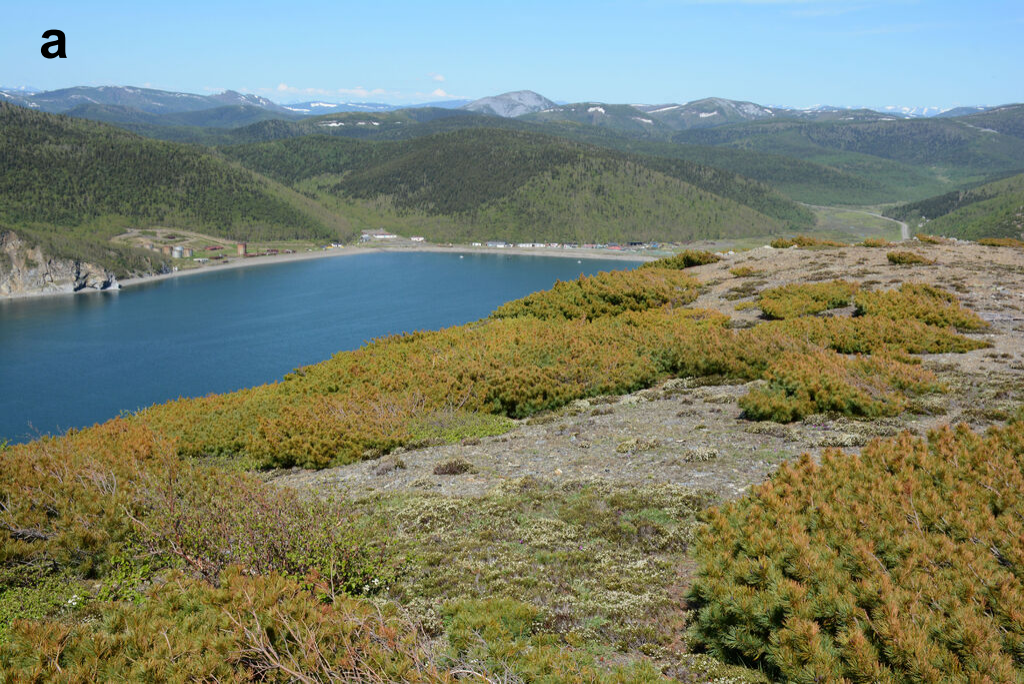
Tundra communities and dwarf *Pinus pumila* (Pall.) Regel clumps are in the foreground, the coastal hills (140 m a.s.l.), dark-coniferous forests on the valley slopes are visible in the background (Ayan Settl. surroundings, east coast of Ayanskaya Bay).

**Figure 2b.**
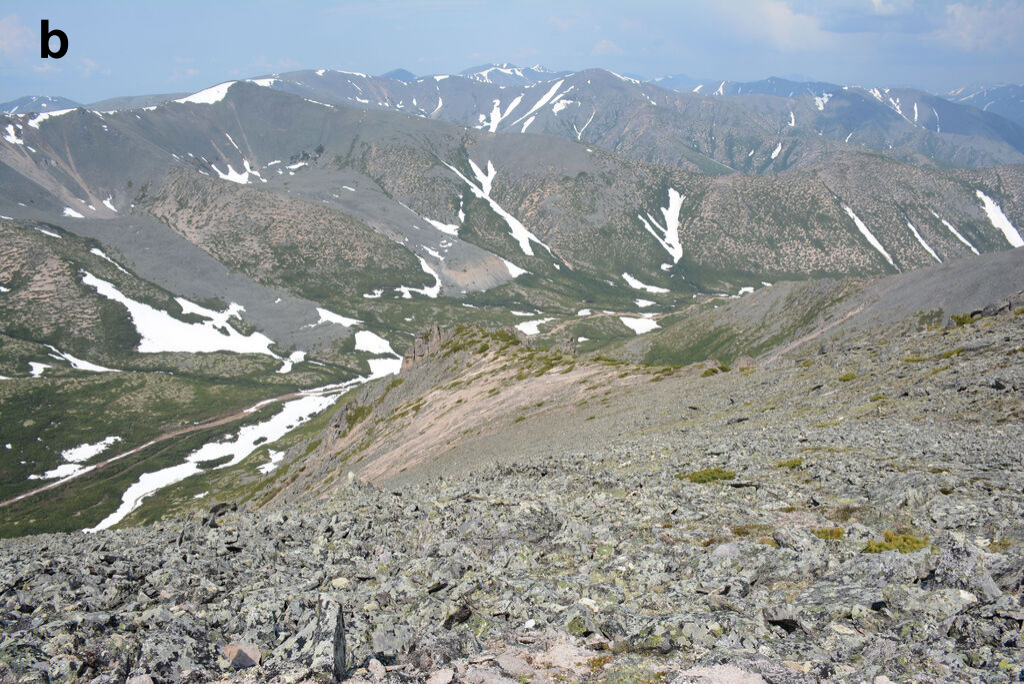
Rock fields on mountain ranges (1400–1500 m a.s.l.) in axial part of Dzhugdzhur, green spots – *Pinus pumila* (Pall.) Regel scattered clumps and sizable thickets.

**Figure 2c.**
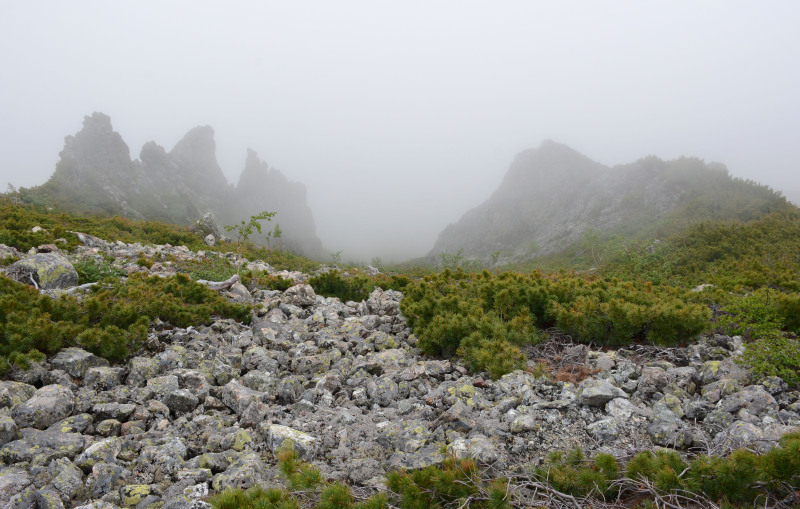
Rock fields with dwarf *Pinus pumila* (Pall.) Regel in upper mountain belt of Pribrezhnyi Range (780 m a.s.l.).

**Figure 2d.**
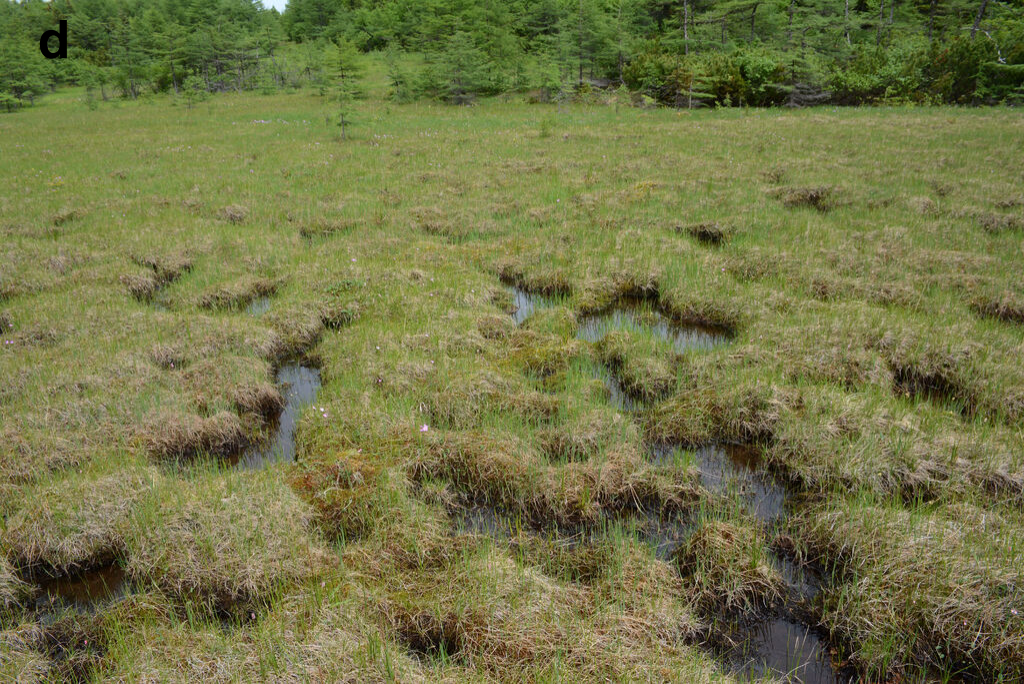
Eutrophic hypnaceous swamp developed over limestone, with sluggishly-flowing waters (91 m a.s.l.) (northwest of Ayan Settlement).

**Figure 3a.**
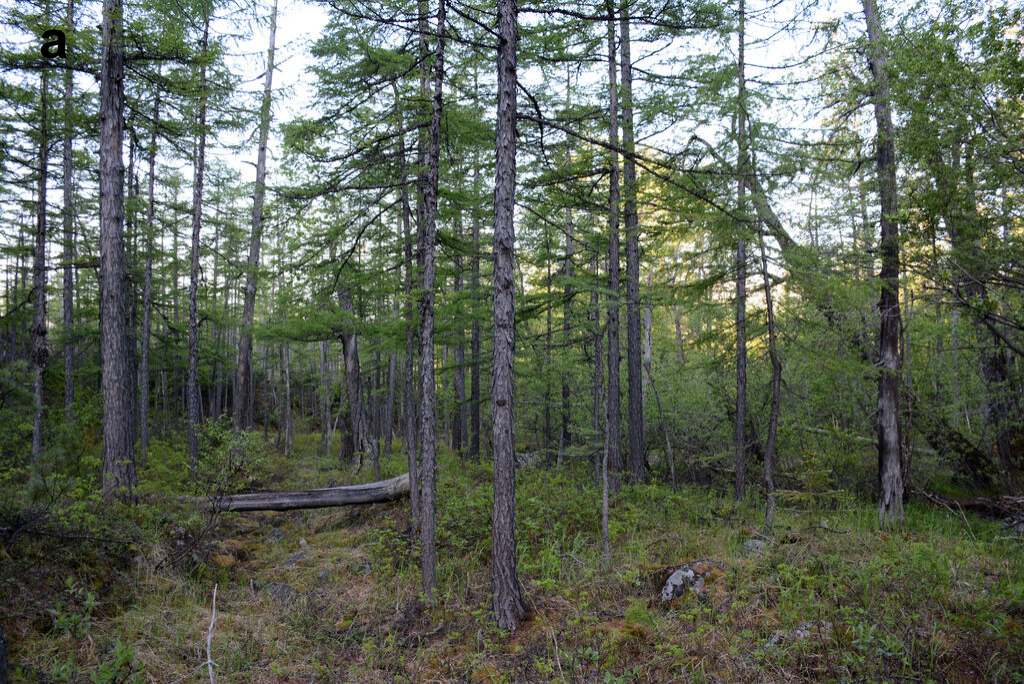
*Larix cajanderi* Mayr forest in the valley (300 m a.s.l.), spurs of Dzhugdzhur Range

**Figure 3b.**
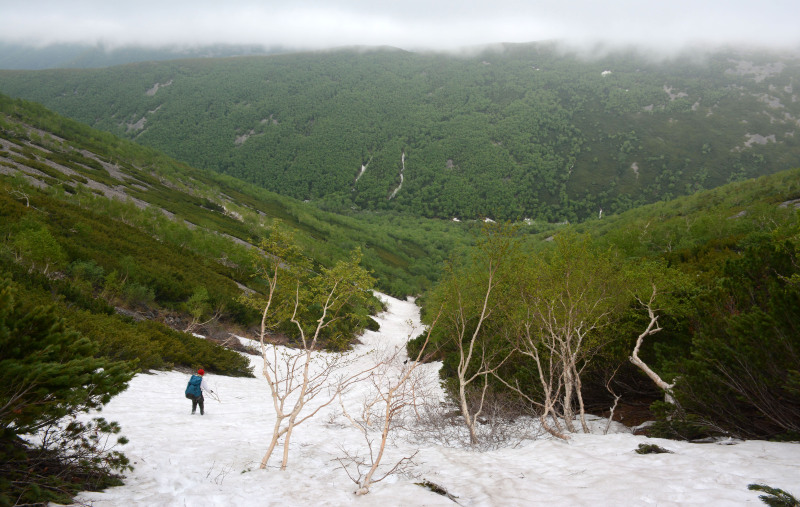
Steep slope to the river valley along a deep creek: *Betula lanata* scattered trees above, surrounded by intermingled strips of *Pinus pumila* (Pall.) Regel thickets and *Betula lanata* (Regel) V.N. Vassil. crooked forest on slope (Pribrezhnyi Range, Unych’ya River valley, the view from ca. 700 m a.s.l.)

**Figure 5a.**
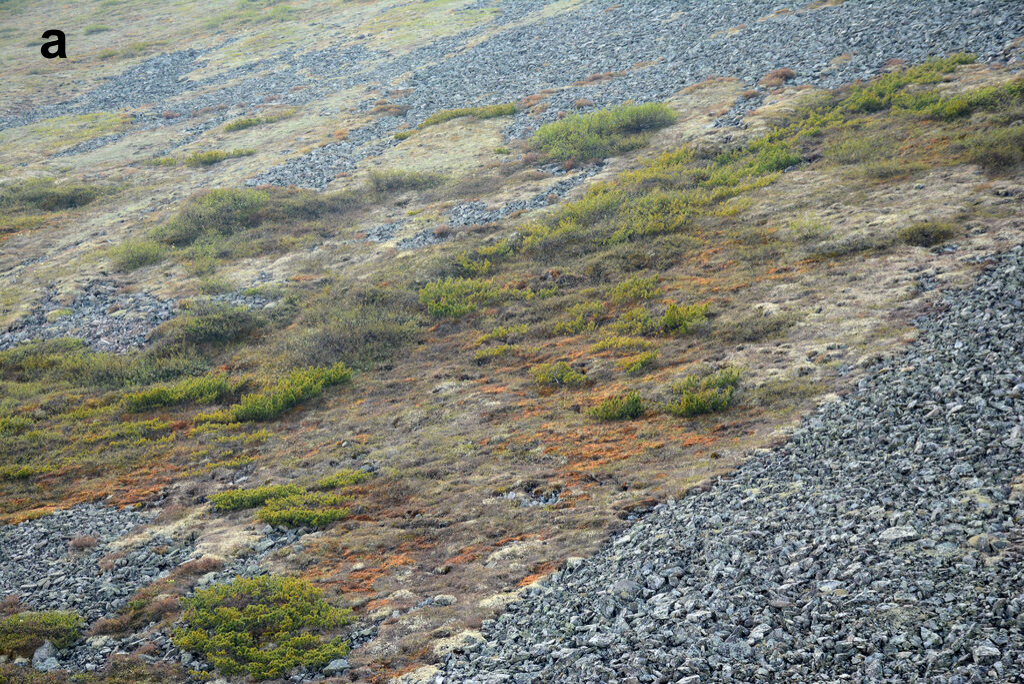
Alpine belt with sparse vegetation represented by dwarf shrub-lichen tundra and *Sphagnum lenense* H. Lindb. ex Pohle mats over percolating water openings, surrounded by rock fields (axial part of Dzhugdzhur Range, locality 11, 1148 m a.s.l.).

**Figure 5b.**
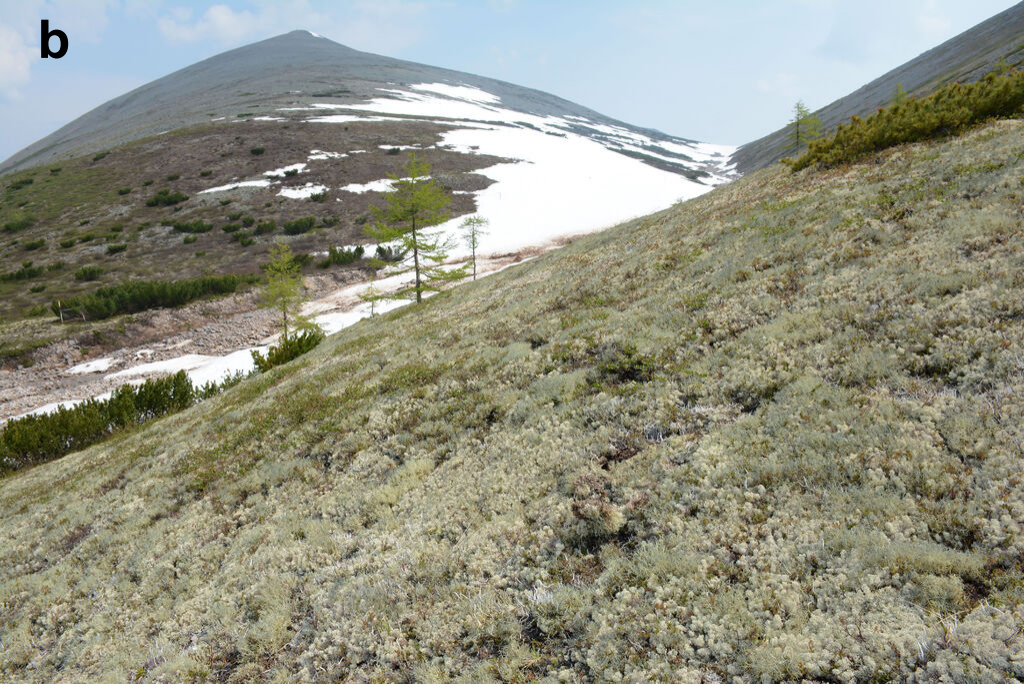
Pure lichen tundra turning into dwarf shrub-lichen tundra (1200 m a.s.l.) with scattered dwarf *Pinus pumila* (Pall.) Regel clumps in the background (axial part near the pass over main ridge of Dzhugdzhur Range).

**Figure 5c.**
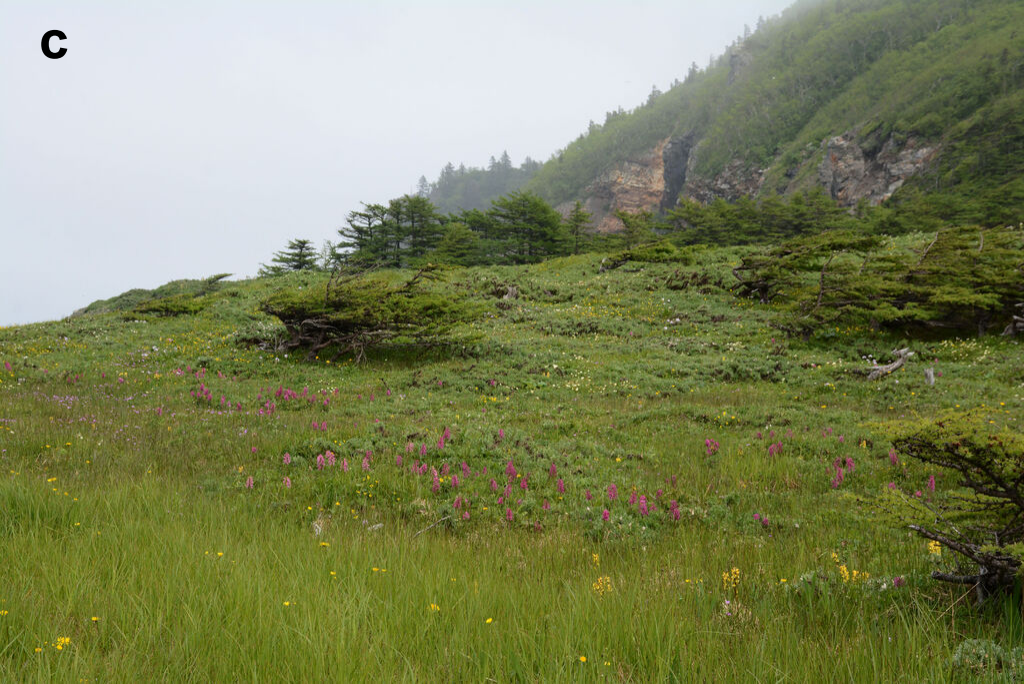
Grass tundra-like community transformed into dwarf-shrub thickets surrounded with ‘flag formed’ trees of *Larix cajanderi* Mayr in a saddle near to the seacoast (100 m a.s.l.), under severe wind conditions (Ayan Settl. surroundings).

**Figure 5d.**
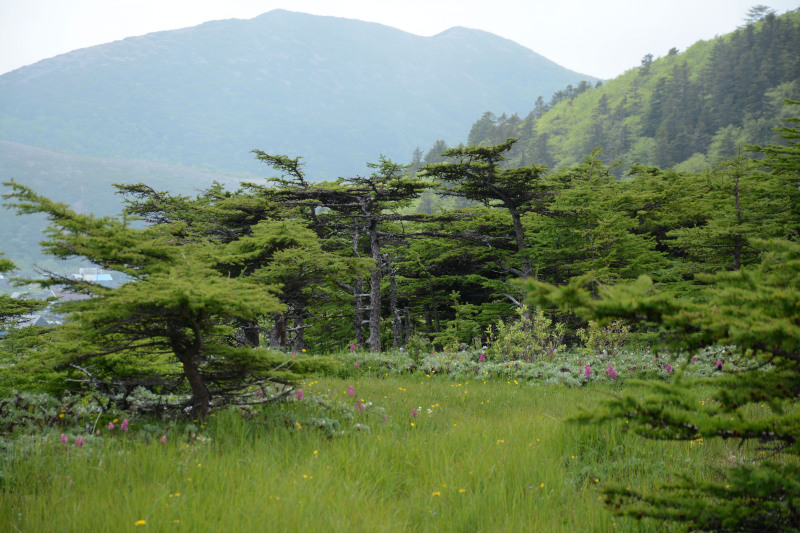
‘Flag-formed’ trees of *Larix cajanderi* Mayr amongst grass tundra-like community in a saddle near the seacoast (100 m a.s.l.), with *Picea ajanensis* Fisch. ex Carrière – *Betula lanata* (Regel) V.N. Vassil. forest in the background (Ayan Settl. surroundings).

**Figure 8a.**
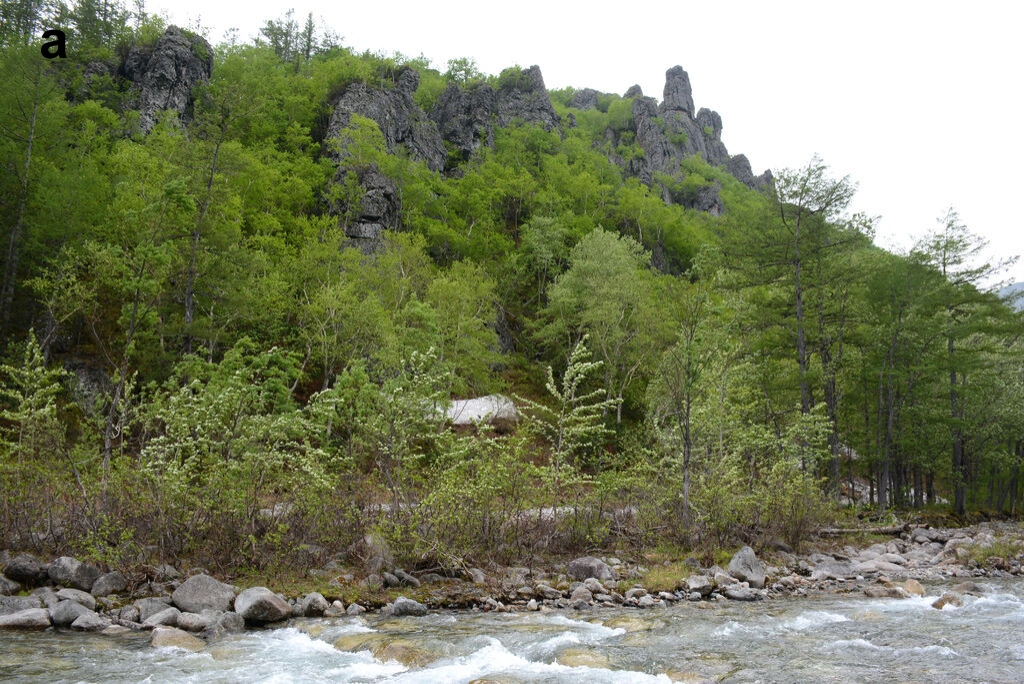
Large N-facing conglomerate cliffs (129–154 m a.s.l.) with scattered *Betula lanata* along riverside (Pribrezhnyi Range, Unych’ya River Valley).

**Figure 8b.**
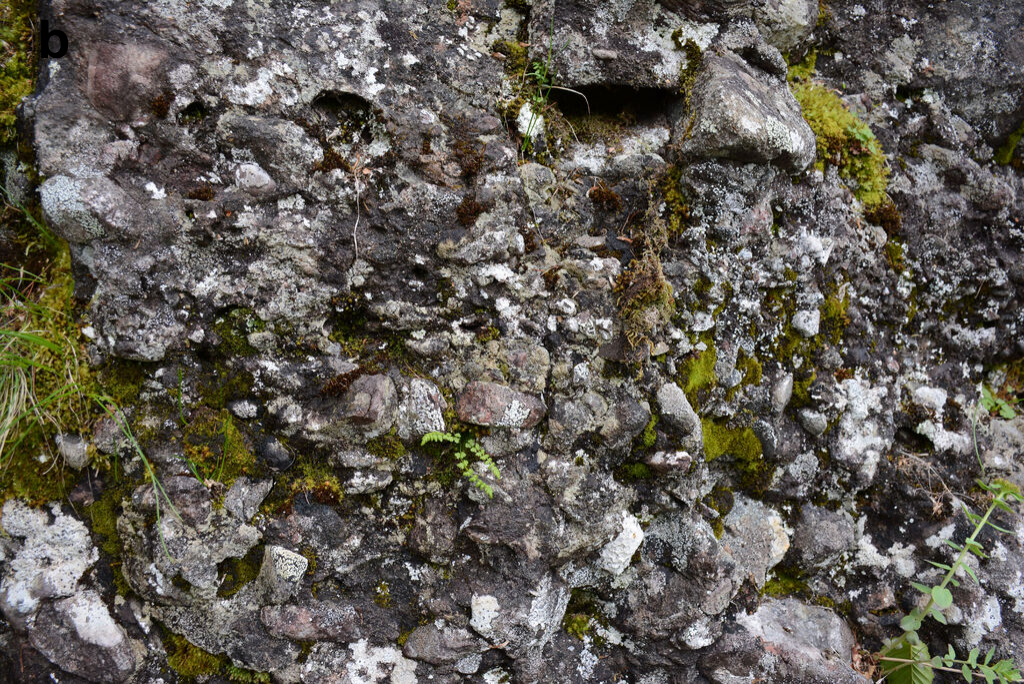
Close-up picture of conglomerate rocks (129 m a.s.l.) depicted in Figure 8a.

**Table 1.** The list of collection localities.

**Locality**	**Locality description**	**Coordinates**	**Elevation, m a.s.l.**
1	Small hill south of Ayan Settl., subalpine belt with many *Duschekia fruticosa* and *Pinus pumila* clumps on the slope ca. 30°	56°27'24.0"N 138°10'16.2"E	11
2	Small hill south of Ayan Settl., upper reaches of subalpine belt near the top of the hill, covered mostly with the mosaic of alpine and subalpine scattered vegetation	56°27'09.5"N 138°10'25.1"E	141
3	Eastern spur of Dzhugdzhur Range, wide valley of small stream (left tributary of Aldoma River) with flood plain vegetation by *Populus suaveolens* and *Salix udensis*, surrounded by *Larix* forest with some clumps of *Pinus pumila* in the understorey	56°36'39.1"N 137°34'14.5"E	294
4	Eastern spur of Dzhugdzhur Range, wide valley of small stream (left tributary of Aldoma River) with flood plain vegetation by *Populus suaveolens* and *Salix udensis*, surrounded by *Larix* forest with some clumps of *Pinus pumila* in the understorey	56°36'53.4"N 137°34'45.8"E	377
5	Eastern spur of Dzhugdzhur Range, gravelly barrens on steep slope to stream valley	56°36'57.8"N 137°34'51.9"E	418
6	Upper reaches of Tugorma River near the main ridge of Dzhugdzhur Range, mosaic of *Duschekia fruticosa* and *Pinus pumila* clumps and mountain tundra vegetation on N-facing slope to stream	56°39'18.3"N 137°15'33.9"E	904
7	Upper reaches of Tugorma River near the main ridge of Dzhugdzhur Range, alpine vegetation with many rocky outcrops and gravelly barrens on steep NE-facing slope to stream	56°38'47.9"N 137°15'30.7"E	1261
8	Upper reaches of Tugorma River near the main ridge of Dzhugdzhur Range, alpine vegetation with many rocky outcrops and gravelly barrens on N-facing slope along small spur	56°38'44.0"N 137°15'25.9"E	1328
9	Upper reaches of Tugorma River near the main ridge of Dzhugdzhur Range, alpine vegetation with many rocky outcrops and gravelly barrens moistened by neutral to basic reaction percolate water on steep N-facing slope	56°38'28.7"N 137°15'23.6"E	1484
10	Upper reaches of Tugorma River near the main ridge of Dzhugdzhur Range, scattered alpine vegetation with many rocky outcrops and gravelly barrens moistened by neutral to basic reaction percolate water on steep W-NW-facing slope	56°38'24.0"N 137°15'09.7"E	1478
11	Area near the pass over main ridge of Dzhugdzhur Range, alpine belt with sparse vegetation represented by dwarf shrub-lichen tundra and *Sphagnum lenense* H. Lindb. ex Pohle mats over percolated water openings, surrounded by gravelly barrens	56°37'47.1"N 137°14'25.6"E	1148
12	Area near the pass over main ridge of Dzhugdzhur Range, its western macro-slope (Arctic Ocean Basin), moist mossy tundra near stream	56°37'48.3"N 137°14'03.0"E	1109
13	Area near the pass over main ridge of Dzhugdzhur Range, moist moss-dwarf shrub tundra near stream	56°38'16.3"N 137°14'23.1"E	1139
14	Upper reaches of Sarafanovka River northwards of Ayan Settl., *Picea ajanensis*-*Betula lanata* forest in the valley with some *Salix udensis* near watercourse and with grass cover	56°27'43.7"N 138°12'04.1"E	54
15	Upper reaches of Sarafanovka River north of Ayan Settl., mostly *Picea ajanensis* forest in narrow valley	56°27'32.7"N 138°13'01.5"E	143
16	Saddle in the hilly landscape near sea coast north of Ayan Settl., windy community with low grasses and shrubs	56°27'58.7"N 138°12'01.0"E	105
17	Pribrezhnyi Range, Unych’ya River valley, large riverside SE-S-facing cliffs	56°25'26.4"N 137°58'16.3"E	82
18	Pribrezhnyi Range, Unych’ya River valley, large N-facing conglomerate cliffs with scattered *Betula lanata* along riverside	56°25'07.3"N 137°56'54.7"E	129–154
19	Pribrezhnyi Range, its northern part, small mountain ca. 850 m alt. with many rocky outcrops, gravelly barrens and *Pinus pumila* clumps (virtually crooked forest belt)	56°24'36.3.4"N 137°54'33.8"E	780
20	Pribrezhnyi Range, its northern part, Unych’ya River upper course, small streamlet in floodplain forest	56°23'59.6"N 137°55'39.0"E	200
21	Pribrezhnyi Range, its northern part, Unych’ya River upper course, small waterfall in deep canyon at the right side of the river	56°23'43.7"N 137°55'19.4"E	235
22	Pribrezhnyi Range, its northern part, Unych’ya River middle course, shaded cliffs along riverside	56°25'07.34"N 137°56'43.65"E	119
23	Pribrezhnyi Range, its northern part, Unychia River middle course, *Picea* forest with some *Betula lanata* trees in the valley	56°25'10.03"N 137°56'59.29"E	118
24	Ayan Settl. surroundings, northwestward of the settlement, steep N-NE-facing slope to sea with many limestone outcrops surrounded by tundra-like communities (developed mostly due to severe wind conditions)	56°29'36.5"N 137°07'58.9"E	34
25	Ayan Settl. surroundings, northwest of the settlement, open *Larix* forest in small stream valley with many limestone boulders in the streambed	56°29'37.7"N 138°07'53.3"E	22
26	Ayan Settl. surroundings, northwest of the settlement, eutrophic hypnaceous swamp developed over limestone, with sluggishly flowing waters	56°29'25.5"N 138°07'24.3"E	91
27	Ayan Settl. surroundings, southern coast of Ayanskaya Bay, basic sedimentary N-facing rocky outcrops along seacoast	56°27'32.6"N 138°09'49.1"E	5
28	Ayan Settl. surroundings, western coast of Ayanskaya Bay, basic sedimentary N-facing rocky outcrops along seacoast	56°27'07.4"N 138°09'09.2"E	5

**Table 2.** The list of floras involved in the comparison.

**№**	**Abbreviations of floras**	**Explanation of the abbreviation** **, literature sources**	**Approximate coordinates**
1	ANIV	Aniva Bay and Aniva Peninsula in Sakhalin Island (Bakalin et al. 2012)	46°30′N 142°30′E
2	ANN	Annachag Range in Magadan Province (Bakalin et al. 2019)	62°00′N 149°30′E
3	BAK	Bakening Volcano and adjacent mountains in East Kamchatka (Bakalin et al. 2019)	54°00′N 158°00′E
4	BYSTR	Bystrinsky Nature Park (Klimova 2015)	56°00′N 158°30′
5	CHAN	Changbaishan Mts. in north-east China (Söderström 2000)	42°00′N 128°00′E
6	COM	Commander Islands (Bakalin 2005, Bakalin 2009a)	55°00′N 166°00′E
7	DEO	Deokgyu Mts. in Korean Peninsula (Choi et al. 2013)	36°00′N 127°30′E
8	Ic	central Iturup Island, from Reidovo to Goryachy Klyuch, Kuril Islands (Bakalin 2009b, Bakalin et al. 2019)	45°00′N 149°00′E
9	In	northern Iturup Island (Bakalin et al. 2019)	45°30′N 148°30′E
10	Is	south Iturup Island, south of Ic, Kuril Islands (Bakalin 2009b, Bakalin et al. 2019)	44°30′N 147°00′E
11	KUN	Kunashir Island, Kuril Islands (Bakalin et al. 2009, Bakalin 2009b, Bakalin et al. 2019)	44°00′N 146°00′E
12	LANZ	Lanzhinskiye Mts. in North Okhotiya (Omelko et al. 2010)	59°30′N 143°30′E
13	NAB	Nabilsky Range of Sakhalin (Bakalin et al. 2012)	51°00′N 143°00′E.
14	NAL	Nalychevo Nature Park, Nalycheva River valley and adjacent volcanoes in East Kamchatka (Bakalin and Klimova 2018)	53°30′N 159°00′E
15	NH	Shiretoko, Nemuro Abashiri Peninsulas, Hokkaido Island (Yamada 2007)	44°00′N 145°00′E
16	NSA	United floras of northern part of Sikhote-Alin Mts., north of 47º N (Bakalin 2015, unpublished data)	48°53′N 138°02′E
17	OLS	Ol’skoye Basalt Plateau (Bakalin et al. 2019)	60°30′N 151°00′E
18	PAR	Paramushir Island, Kuril Islands (Bakalin and Cherdantseva 2006)	51°30′N 156°00′E
19	RISH	Rishiri Island, opposite to western cost of Hokkaido Island (Ooishi and Yamada 2008)	45°00′N 141°00′E
20	SCHM	Schmidt Peninsula in Sakhalin Island (Bakalin et al. 2012)	54°00′N 142°30′E
21	SHIK	Shikotan Island, Kuril Islands (Bakalin et al. 2009)	43°30′N 143°30′E
22	SHIR	Shirakami Mt., Aomori Prefecture, Honshu Island (Higuchi and Furuki 1996)	40°30′N 140°00′E
23	SSA	United floras of southern part of Sikhote-Alin Mts., south of 47º N (Bakalin 2008, unpublished data)	43°14′N 133°43′E
24	VMG	East-Manchurian Mts. (Kedrovaya Pad’ Nature Reserve, mountains west of Razdolnaya River valley, Sinyaya Mt. (Bakalin et al. 2017, unpublished data)	43°07′N 131°28′E
25	AYAN	Ayan Settlement surroundings, Dzhugdzhur Range, Pribrezhnyi Range (data from the present account)	56°27'N 138°12'E

**Table 3.** Normalized values of DCA for each compared flora

**Floras**	**Axis 1**	**Axis 2**	**Axis 3**
In	0.521978	0.297989	0.598394
Ic	0.485348	0.411335	0.626506
Is	0.507326	0.378428	0.668675
SHIK	0.6337	0.334552	0.616466
KUN	0.532967	0.374771	0.728916
ANIV	0.556777	0.508227	0.592369
NAB	0.371795	0.506399	0.52008
PAR	0.315018	0.416819	0.821285
SCHM	0.408425	0.506399	0.550201
LANZ	0.336996	0.519196	0.552209
NAL	0.300366	0.440585	0.47992
BAK	0.272894	0.466179	0.554217
ANN	0.17033	0.500914	0.62249
OLS	0	0.61426	0.46988
DEO	1	0.531993	0.608434
CHAN	0.580586	0.722121	1
SHIR	0.782051	0	0.528112
NH	0.684982	0.40585	0
RISH	0.635531	0.223035	0.634538
BYSTR	0.221612	0.435101	0.742972
COM	0.289377	0.433272	0.843373
NSA	0.465201	0.66362	0.419679
SSA	0.631868	0.773309	0.459839
VMG	0.789377	1	0.600402
AYAN	0.326007	0.678245	0220884

**Table 4.** The mean distances between Ayan flora and other floras involved in the analysis. The font in bold indicates that distances are less than the mean value in the data samples.

**Floras**	**Distances**
**NSA**	**0.243**
**NAB**	**0.348**
**NAL**	**0.352**
**LANZ**	**0.368**
**SCHM**	**0.380**
**BAK**	**0.399**
**SSA**	**0.400**
**OLS**	**0.415**
ANN	0.466
ANIV	0.469
NH	0.502
Ic	0.511
Is	0.569
In	0.571
BYSTR	0.585
SHIK	0.608
KUN	0.627
PAR	0.655
COM	0.670
VMG	0.680
RISH	0.689
DEO	0.791
CHAN	0.821
SHIR	0.873
